# Peptide ligands targeting FGF receptors promote recovery from dorsal root crush injury via AKT/mTOR signaling

**DOI:** 10.7150/thno.62525

**Published:** 2021-11-02

**Authors:** Ying Zhao, Qiang Wang, Chen Xie, Yuling Cai, Xue Chen, Yuhui Hou, Liu He, Jianping Li, Min Yao, Shuangxi Chen, Wutian Wu, Xiaojia Chen, An Hong

**Affiliations:** 1 Institute of Biomedicine & Department of Cell Biology, College of Life Science and Technology, Jinan University; Guangdong Province Key Laboratory of Bioengineering Medicine; Guangdong Provincial biotechnology drug & Engineering Technology Research Center; National Engineering Research Center of Genetic Medicine, Guangzhou, Guangdong, 510632, P. R. China.; 2 Guangdong-Hong Kong-Macau Institute of CNS Regeneration, Ministry of Education CNS Regeneration Collaborative Joint Laboratory, Jinan University, Guangzhou, Guangdong 510632, China.; 3 School of Pharmaceutical Sciences, Health Science Centre, Shenzhen University, Shenzhen, 518060, China.

**Keywords:** Peptides, Fibroblast growth factor receptor, Dorsal root ganglia, Nerve regeneration, Dorsal root crush injury

## Abstract

**Background:** Fibroblast growth factor receptors (FGFRs) are key targets for nerve regeneration and repair. The therapeutic effect of exogenous recombinant FGFs *in vivo* is limited due to their high molecular weight. Small peptides with low molecular weight, easy diffusion, low immunogenicity, and nontoxic metabolite formation are potential candidates. The present study aimed to develop a novel low-molecular-weight peptide agonist of FGFR to promote nerve injury repair.

**Methods:** Phage display technology was employed to screen peptide ligands targeting FGFR2. The peptide ligand affinity for FGFRs was detected by isothermal titration calorimetry. Structural biology-based computer virtual analysis was used to characterize the interaction between the peptide ligand and FGFR2. The peptide ligand effect on axon growth, regeneration, and behavioral recovery of sensory neurons was determined in the primary culture of sensory neurons and dorsal root ganglia (DRG) explants *in vitro* and a rat spinal dorsal root injury (DRI) model *in vivo*. The peptide ligand binding to other membrane receptors was characterized by surface plasmon resonance (SPR) and liquid chromatography-mass spectrometry (LC-MS)/MS. Intracellular signaling pathways primarily affected by the peptide ligand were characterized by phosphoproteomics, and related pathways were verified using specific inhibitors.

**Results:** We identified a novel FGFR-targeting small peptide, CH02, with seven amino acid residues. CH02 activated FGFR signaling through high-affinity binding with the extracellular segment of FGFRs and also had an affinity for several receptor tyrosine kinase (RTK) family members, including VEGFR2. In sensory neurons cultured *in vitro*, CH02 maintained the survival of neurons and promoted axon growth. Simultaneously, CH02 robustly enhanced nerve regeneration and sensory-motor behavioral recovery after DRI in rats. CH02-induced activation of FGFR signaling promoted nerve regeneration primarily via AKT and ERK signaling downstream of FGFRs. Activation of mTOR downstream of AKT signaling augmented axon growth potential in response to CH02.

**Conclusion:** Our study revealed the significant therapeutic effect of CH02 on strengthening nerve regeneration and suggested a strategy for treating peripheral and central nervous system injuries.

## Introduction

Neurological disorders are recognized as a leading cause of death and disability worldwide. The nervous system is vulnerable to various disorders and can be damaged by many factors, including trauma, infections, degeneration, structural defects, tumors, blood flow disruption, and autoimmune disorders. Findings from the Global Burden of Diseases, Injuries, and Risk Factors Study (GBD) in 2015 and 2016 1, 2 showed that neurological disorders are the largest cause of disability, and their contribution to the overall burden of all health conditions is increasing. Once injured, mature neurons in the mammalian central nervous system (CNS) fail to regenerate their axons due to poor intrinsic regenerative capacity coupled with the hostile CNS environment 3-6. The failure of axon regeneration by damaged adult CNS neurons and to rebuild functional connections cause permanent disabilities in individuals with spinal cord injury or stroke 7. In contrast, mammalian peripheral nervous system (PNS) neurons retain the ability to self-repair and reactivate intrinsic growth programs following injury and better regenerate than those in the CNS 3, 8. However, severe damage to peripheral nerves can also cause permanent neurological deficits, including failure to reinnervate and chronic pain development, resulting in severe disability and decreased quality of life 9.

The current repair strategies for healing peripheral nerve injuries include end-to-end repair, nerve grafting, conduit implantation, and stem cell therapy 10, 11. However, these methods are highly dependent on the repairability of the neurons themselves. Activating the repair mechanism of damaged neurons can promote the recovery of neuronal function with conventional treatment methods and improve patient prognosis. Growth factors (GFs) are a family of proteins that regulate biological development and neural function, including regulating the survival of neurons, enhancing synaptic function recovery, and boosting axon growth and remodeling 12, 13. In multiple drug treatment methods, the administration of exogenous GFs is an emerging and versatile therapeutic strategy for enhancing peripheral nerve regeneration and functional recovery 14, 15. Furthermore, GFs alone or in combination with other methods can lead to better neural circuit reconstruction and functional recovery from spinal cord injury (SCI) 16. Some GFs have been well investigated and employed in preclinical trials 14, such as brain-derived neurotrophic factor (BDNF) 17, fibroblast GF 2 (FGF2) 18, and neurotrophin-3, (NT-3) 19, 20.

The generation of a functional nervous system involves a variety of steps controlled by a few families of extracellular signaling molecules during neural development. Of the many molecules, FGFs and their receptors, highly conserved among the animal kingdom, appear to have the most widespread and best-documented roles in generating cellular diversity and morphological complexity of the nervous system 21-23. Besides their documented role during the early steps of neural primordium structure and neural progenitor cell proliferation, FGFs and their receptors also play an essential role in the latter stages of neural development, including neuron migration, axon outgrowth, axon navigation, and synapse formation 21, 24, 25. In case of nervous system damage, the developmental process is reactivated 21. Many studies have demonstrated that key factors involved in axon outgrowth during neural development may also promote regeneration of injured adult neurons 26. Members of the FGF family, especially FGF2, regulate neuroprotection and repair in response to nerve injury 21. In animal models, studies have shown that neuronal regeneration in response to transient ischemia or traumatic brain injury requires FGF2 and FGFR1 27, 28. Exogenous FGF2, either alone or in combination with other factors such as BDNF or endothelial GF (EGF), also promotes robust neuronal regeneration in neurodegenerative diseases, such as Huntington's disease 29. Moreover, the protection against neuronal death is also an essential role of FGF2 in neurodegenerative diseases 29, 30. Thus, exogenous FGF application is an effective therapeutic strategy for strengthening nerve repair. FGFRs are important targets for nerve regeneration and functional recovery. An in-depth study of the role of FGF in the occurrence and development of neurological diseases will help to improve treatment methods targeting FGFR.

There are, however, several limitations of administering exogenous FGFs, including immunogenicity, limited diffusion, and the inability to cross the blood-spinal cord barrier 14, 16. Further, the recombinant protein has a high molecular weight and high production cost 31. Hence, developing alternative small molecule drugs that retain the nerve repair activity of FGF but overcome its limitations would be an effective strategy for boosting nerve regeneration and functional recovery. Compared to recombinant proteins, small peptides with characteristics, such as small molecular weight, low production cost, easy diffusion, and low immunogenicity, are the best candidates for potential small molecule drugs. The beneficial effects of small peptides can be assessed in mammalian dorsal root ganglion (DRG) neurons that project both a peripheral axonal branch into peripheral nerves and a central axonal branch through the dorsal root into the spinal cord, representing a favorable system for studying mammalian axon regeneration 3, 8.

Herein, using a phage display peptide library, we identified a novel FGFR-targeting small peptide, CH02, that exerted a significant therapeutic effect on strengthening nerve regeneration.

## Results

### Screening and identification of small peptides targeting FGFR2

**Figure 1:**
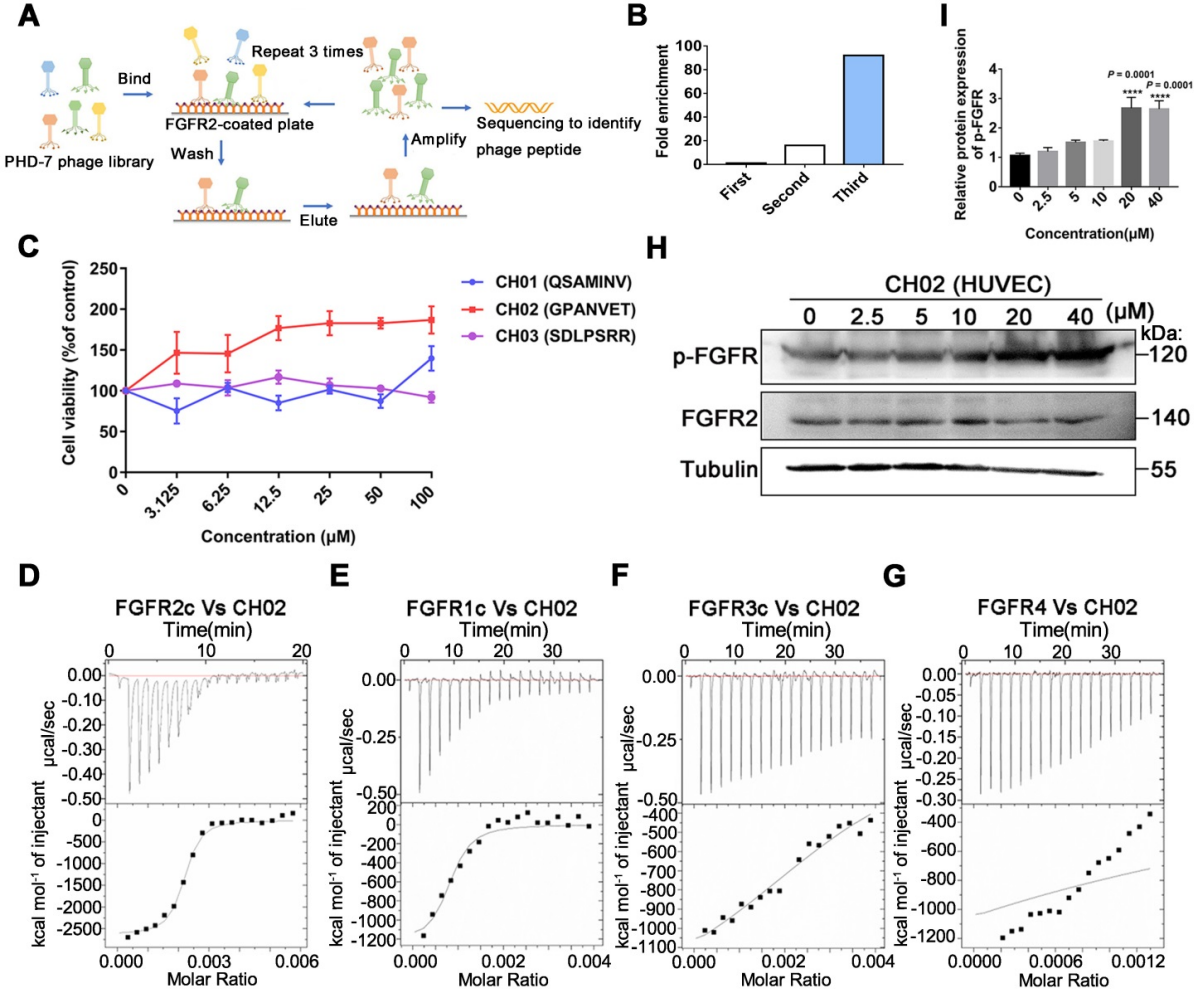
** Screening of FGFR2-targeted small peptides using a phage display peptide library. (A)** Illustration of the library screening steps. The phage library was incubated on a plate precoated with a recombinant FGFR2 extracellular domain protein, the unbound phage was washed, and the specifically bound phage was eluted. The eluted phages were amplified and used as input for the next round of biopanning. After three rounds, individual clones were sequenced to identify the phage peptides. **(B)** After each screening round, phage recovery (%) was assessed by phage titer. Bars represent multiple changes in the phage recovery in each round compared to the recovery in the first round. **(C)** Cell viability assays of FGFR2-overexpressing HEK293 cells incubated with CH01, CH02, or CH03 peptides at a concentration gradient for 48 h. **(D-G)** ITC analysis of FGFR1c, FGFR2c, FGFR3c, or FGFR4 binding to the CH02 peptide. Top panels show the raw data of the heat pulses resulting from FGFR1c, FGFR2c, FGFR3c, or FGFR4 titration. Bottom panels show the integrated heat pulses, normalized per mole of injectant, as a function of the molar ratio (CH02 peptide concentration/recombinant protein concentration). These binding curves were best fitted to a single-site binding model. **(H)** Western blot analysis of phosphorylated FGFR expression in cultured human umbilical vein endothelial cells (HUVECs) treated with CH02 in a concentration gradient. **(I)** Quantification of p-FGFR expression levels shown in (H). n = 3 independent experiments. The relative protein expression level was quantified after normalization to FGFR2 (***p < 0.001 by one-way ANOVA with Dunnett's test, compared to the control group). Values are means ± SEM.

Considering the limitations of applying exogenous large molecular weight recombinant FGFs to enhance nerve repair, we screened small peptides targeting FGFRs using the phage display peptide library. FGFRs are receptor tyrosine kinases (RTKs) and consist of four members (FGFR1, FGFR2, FGFR3, and FGFR4) involved in various biological processes, such as developmental regulation and tissue repair 32. We used the recombinant protein FGFR2IIIc extracellular domain as the target receptor and a Ph.D.-7 library displaying random 7-mer peptide sequences for biopanning (Figure 1A). We kept the number of phage inputs (2.0 × 10^11^) unchanged, but the coating concentration of FGFR2 protein was gradually reduced (10, 5, 2.5 μg). Phage recovery (%) increased 92-fold after 3 rounds compared to the first round (Figure 1B and Table 1). Next, individual phage clones randomly selected from the third round were sequenced, and three small peptides (CH01, CH02, and CH03) were identified. Subsequently, a cell proliferation assay was performed to investigate the biological activity of these peptides and the CH02 peptide, exhibiting the most significant cell proliferation activity (Figure 1C), was selected for further investigation. Isothermal titration calorimetry (ITC) assay was conducted to verify the binding affinity between the CH02 peptide and FGFR1, FGFR2, FGFR3, and FGFR4 (Figure 1D-G). However, the CH02 peptide showed a higher binding affinity with FGFR1 and FGFR2 than FGFR3 and FGFR4. Immunoblotting revealed that CH02 upregulated FGFR phosphorylation in a concentration-dependent manner (Figure 1H-I). In summary, using a phage peptide library, we identified a novel FGFR-targeting small peptide CH02 with seven amino acid residues that induced a remarkable proliferation effect. CH02 exhibited a high binding affinity for FGFR1 and FGFR2 and simultaneously activated the FGFR signal.

### Molecular docking and dynamic simulation analysis of the binding mode between CH02 and FGFR2

**Figure 2:**
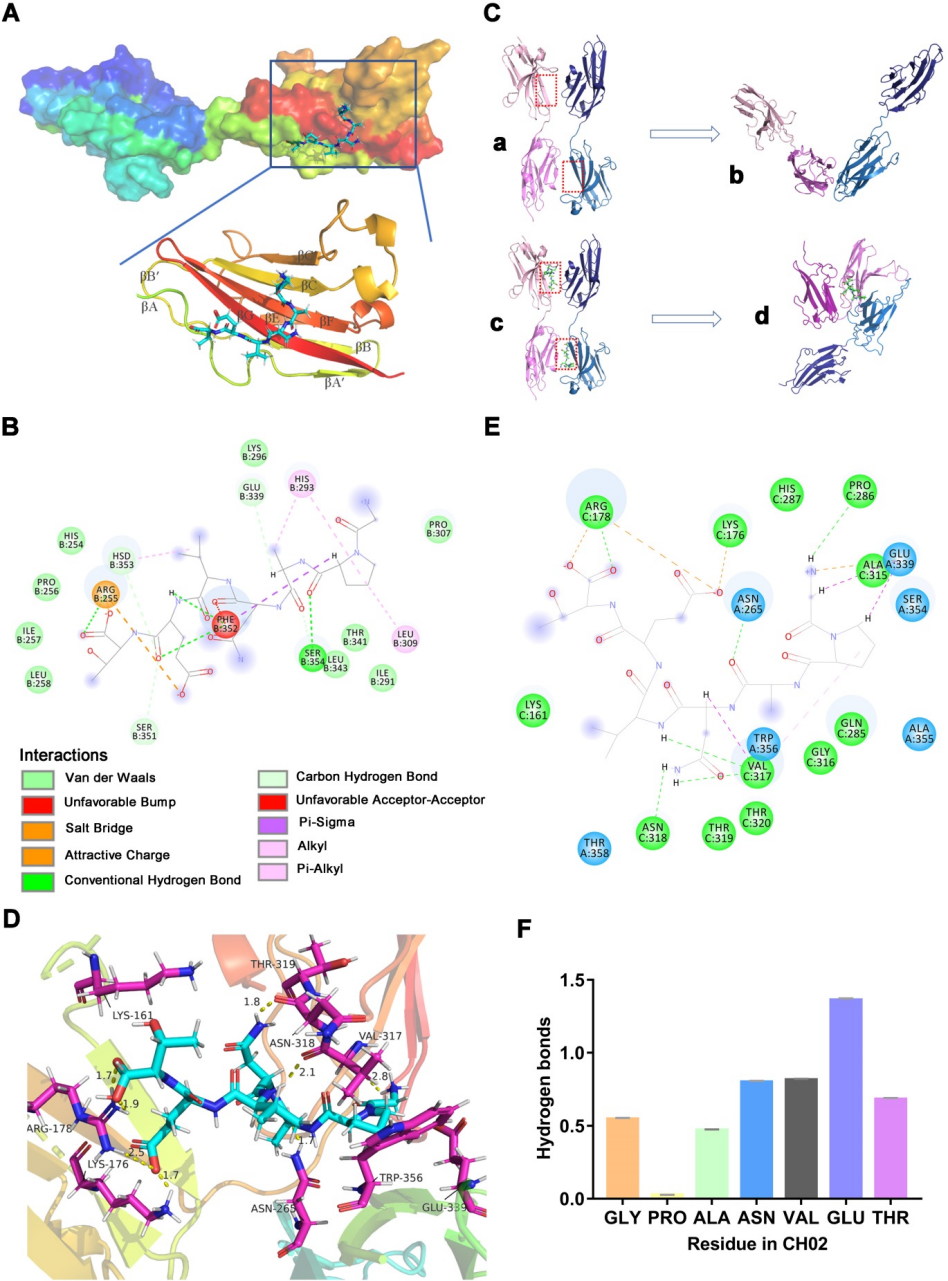
** Molecular docking and MDS analysis of CH02 interactions with FGFR2 receptors. (A)** Binding pattern between CH02 and the FGFR2 extracellular segment. The surface model represents the FGFR2 extracellular domain, the cartoon model shows a partial enlargement of the D3 area, and the stick model shows the CH02 peptide. **(B)** Two-dimensional binding mode shows noncovalent interaction between CH02 and FGFR2. Each balloon displays different residues, and non-covalent bonds are shown as dashed lines in different colors. **(C)** MDS results of two CH02-FGFR2 docking complexes (CH02-FGFR2:CH02-FGFR2) renamed Complex-2. The remaining components (FGFR2:FGFR2) after removing the CH02 ligand from Complex-2 were considered the control group and called Complex-1. (a) Complex-1. (b) Average structure in the last 50 ns of MDS from (a). (c) Complex- 2. (d) Average structure in the last 50 ns of MDS from (c). **(D)** Average structure of the interaction between CH02 and FGFR2 in Complex-2. CH02 is shown as a blue stick model, and partial residues of FGFR2 that interacted with CH02 are shown as purple stick models. **(E)** Types of noncovalent interactions between CH02 and FGFR2 in Complex-2. Each balloon represents different residues, and dashed lines in different colors show noncovalent bonds. **(F)** Number of hydrogen bonds formed by different residues of CH02 and FGFR2 in the simulation process for Complex 2.

Since FGFR2 was selected as the target receptor for CH02 in biopanning, we further explored the binding mode between the CH02 peptide and FGFR2 and conducted molecular docking and MDS. The structure of the FGFR2IIIc extracellular domain was obtained using a homology modeling method. The docking results showed that the best CH02 binding site was in the D3 region of the FGFR2IIIc extracellular domain, containing multiple β-sheet structures, and primarily included βC-βC' and βE-βG segments (Figure 2A). Residues in the CH02 and FGFR2 binding pocket included Arg-255, His-293, Leu-309, Phe-352, and Hsd-353 (Figure S1A). Arg-255 formed a conventional hydrogen bond and an attractive charge with the CH02 peptide, His-293 bound CH02 peptide by two Pi-Alkyl bonds, and Phe-352 bound to multiple residues of the CH02 peptide by two conventional hydrogen bonds and one Pi-Sigma (Figure 2B). Similar to His-293, Leu-309 and Hsd-353 bound the CH02 peptide by Pi-Alkyl, and Hsd-353 bound CH02 by carbon-hydrogen bonding (Figure 2B). Subsequently, an all-atom MDS of the CH02-FGFR2 docking complex was performed using the Gromacs software package in the physiological saline solution at a temperature of 300 K for 100 ns. The root-mean-square deviation (RMSD) values of the heavy atoms of CH02 and FGFR2 were calculated to reflect the structural changes of the complex. The results indicated that the structural framework of the CH02 peptide remained stable throughout the simulation process, while FGFR2 primarily contributed to the framework fluctuation of the CH02-FGFR2 complex (Figure S1B, Supplementary Movie 1). Since the combination of FGF2 and the D3 region of FGFR2 primarily rely on hydrogen bonds 33, we calculated the number of hydrogen bonds formed between the CH02 peptide and FGFR2 during the entire simulation process that remained stable after a fluctuation of approximately 40 ns, suggesting the stability of the binding conformation (Figure S1C).

These results indicated that the CH02 peptide stably bound to the D3 region of the FGFR2 extracellular segment under simulated conditions.

FGFR signals via ligand-dependent dimerization, causing conformational changes in the receptor structure accompanied by activation of the intracellular kinase domain 34. Hence, we determined whether the CH02 peptide affected FGFR2 homodimerization. We placed two CH02-FGFR2 docking complexes (CH02-FGFR2:CH02-FGFR2) close together, one of which was placed upside down, and simulated interaction between two FGFR2 molecules in a physiological state. After removing the ligand CH02 from the two complexes, the remaining components (FGFR2:FGFR2) were considered the control group. The complexes were then subjected to MD simulation for 300 ns in normal saline at a temperature of 300 K (Figure 2C). RMSD was used to evaluate the difference between the complexes containing CH02 (Complex-2) and lacking CH02 (Complex-1) during a 300 ns simulation. The results showed severe structural fluctuations in both complexes in an aqueous solution, while the CH02 peptide framework structure remained stable (Figure S1D). The structural changes of the two complexes were observed by extracting the last 50 ns trajectory of the simulation process and discerning their average structure. Simulation results revealed that the average structures of the two complexes were quite different. In Complex-2, D2 and D3 regions of one FGFR2 were folded close to the D3 region of the other FGFR2, and the CH02 peptide was located at the geometric center of these three domains (Figure 2C, Supplementary Movie 2). However, the average structure of Complex-1 revealed that the two FGFR2s were distant from each other, and only part of the area maintained the close spatial conformation before the simulation (Figure 2C, Supplementary Movie 3).

The number of hydrogen bonds formed between the FGFR2s during the entire simulation process was quantified to understand whether the CH02 peptide affected the interaction between the two FGFR2s. There were no hydrogen bonds between FGFR2 in Complex-1 after 200 ns while Complex-2 sustained hydrogen bond interactions between FGFR2 throughout the simulation period (Figure S1E). To determine the binding stability of the CH02 peptide, the number of hydrogen bonds formed between CH02 and FGFR2 during the simulation was quantified. The results demonstrated that the CH02 peptide sustained hydrogen bond interactions with FGFR2 throughout the simulation period (Figure S1F). The binding mode CH02 in the Complex-2 system was determined by extracting the average structure of the last 50 ns of the simulated trajectory of Complex-2, and the interaction between CH02 and FGFR2 was observed. Our results showed that CH02 bound to the D3 region of these two receptors and to the D2 region of receptor 1 simultaneously with the three domains wrapping around CH02 (Figure 2D). The CH02 peptide and Arg-178 in the receptor 1 D2 region formed two attractive charge interactions by forming a conventional hydrogen bond, and the CH02 peptide also formed a salt bridge with Lys-176. The amino acids of CH02 used to bind to the D3 regions of receptors 1 and 2 were quite different. It formed a conventional hydrogen bond with Pro-289, Asn-318, and Val-317 in the D3 region of receptor 1, a carbon-hydrogen bond and an alkyl bond with Val-317, a carbon-hydrogen bond and an attractive charge with Glu-339 of receptor 2, and a conventional hydrogen bond with Asn-265 (Figure 2E).

Distinct from the binding mode of the docking complex, the CH02 peptide could bind to the βC'-βE segment of the D3 region in both FGFR2 molecules, with high variability among FGFR families 33, suggesting that it may have different effects on the physiological activities of FGFR family members. The number of hydrogen bonds formed between various CH02 peptide residues and two FGFR2s during the simulation process was dynamically quantified. The results showed that Asn-4, Val-5, and Glu-6 in the CH02 peptide contributed more hydrogen bond interactions, while Pro-2 and FGFR2 hardly formed any hydrogen bonds (Figure 2F). Next, we created point mutations in the CH02 amino acids and then tested the core amino acids through which CH02 exerted biological activity using a cell proliferation assay. Consistent with the MD results (Figure 2F), the cell proliferation data showed that Asn-4, Val-5, Glu-6, and Thr-7 were the core CH02 peptide amino acids essential for maintaining its biological activity (Figure S1G-H).

In summary, the CH02 peptide uniquely prompted binding between FGFR2 receptors under simulated conditions.

### CH02 promotes axon outgrowth and survival of primary sensory neurons

**Figure 3:**
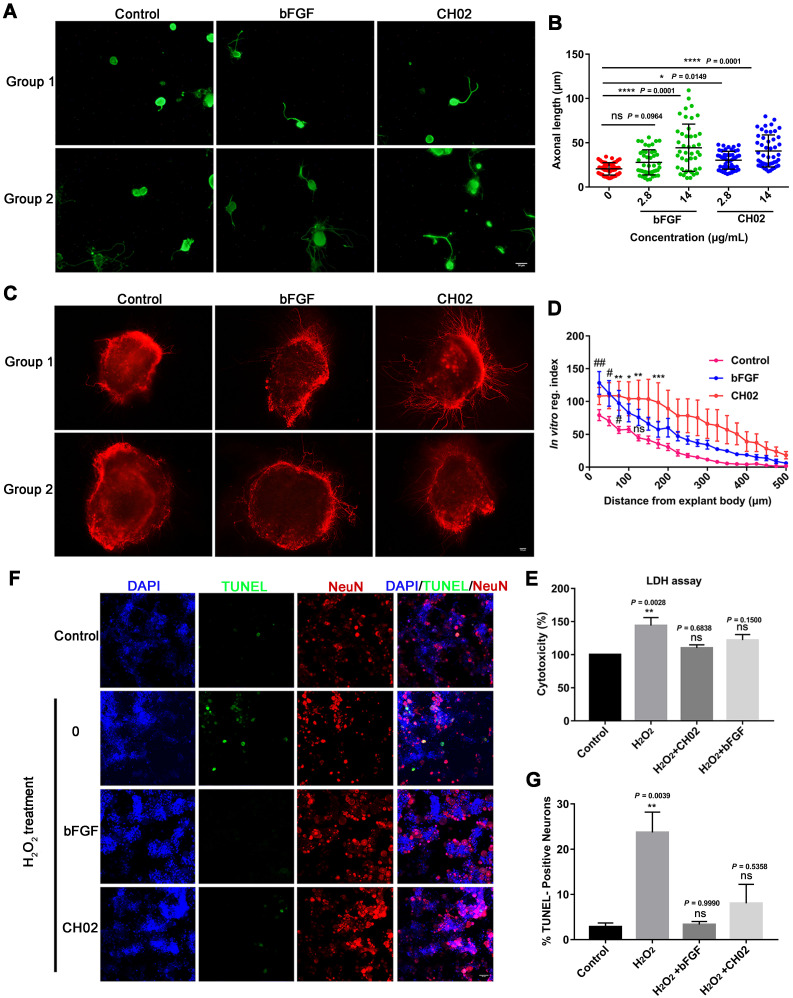
** Effect of the CH02 peptide on axon growth and neuron survival. (A)** Representative images of the CH02 peptide effect on axon growth in adult primary DRG neurons. DRG neurons were obtained from adult Sprague-Dawley rats. After 12-h of culture, neurons were stimulated with CH02 or bFGF for 48 h. Axonal outgrowth was assessed by immunocytochemistry using an antibody against β-tubulin, an axonal marker. Images were acquired from random neurons by fluorescence microscopy. Scale bars = 50 μm. **(B)** Quantification of the longest axon length of each neuron randomly selected in three independent experiments from (A), using Image-Pro Plus 6.0 software (n = 48 for each condition; *p < 0.05, ****p < 0.0001 by one-way ANOVA with Dunnett's test; ns, not significant; mean ± SD). **(C)** Representative images of the CH02 effect on axon growth in *ex vivo* cultured adult DRG explants. The axon is shown in fluorescence (β3-tubulin) images. Scale bars = 100 μm. **(D)** Quantification of axon growth of DRG explants from (C) using the Neurite-J software (n=4 for each condition; *control vs. CH02; #control vs. bFGF; *p < 0.05, **p < 0.01, ***p < 0.001, #*p* < 0.05, ##*p* < 0.01 by two-way ANOVA with Dunnett's test; ns, not significant; mean ± SEM). **(E)** Cell cytotoxicity assay of primary DRG neurons induced by H_2_O_2_. LDH released from damaged cell membranes was detected by an LDH assay kit. The results are expressed as the mean ± SEM (**p < 0.01 by one-way ANOVA with Dunnett's test; ns, not significant, compared with the control group). **(F)** Representative TUNEL staining of apoptotic neurons induced by H_2_O_2_ in the control, CH02, and FGF2 groups. Scale bars = 50 μm. Apoptotic neurons were detected by a TUNEL apoptosis assay kit. TUNEL (green); NeuN, neuronal marker (red); DAPI (blue). **(G)** Quantification of TUNEL-positive neurons from (F) (n = 3 for each condition; **p < 0.01 by one-way ANOVA with Dunnett's test; ns, not significant, compared with the control group). DRG neurons were obtained from adult Sprague-Dawley rats. After 12-h of culture, neurons were pretreated with CH02 or bFGF for 24 h before H_2_O_2_ (500 μM) treatment for 12 h.

We explored the CH02 peptide effect on the axon outgrowth of DRG neurons *in vitro*. Primary DRG neurons were obtained from adult Sprague-Dawley rats. Recombinant human bFGF (146 aa) protein was used as a positive control. The results indicated that CH02 robustly promoted axonal growth of sensory neurons (Figure 3A-B). We further investigated the CH02 effect on axon regeneration in *ex vivo* cultured adult DRG explants. The CH02 peptide strikingly stimulated axonal regeneration of DRG explants (Figure 3C-D). Next, we performed cell cytotoxicity assays of primary DRG neurons induced by H_2_O_2_ to examine whether CH02 enhanced the survival of DRG neurons. Compared to the H_2_O_2_ treatment group alone, CH02 effectively reduced lactate dehydrogenase (LDH) released from the supernatant, suggesting that it protects neurons from apoptosis induced by H_2_O_2_ (Figure 3E). The TUNEL assay further supported the finding that CH02 treatment reduced DRG neuron apoptosis induced by H_2_O_2_ (Figure 3F-G).

Collectively, our data indicated that the CH02 peptide treatment conferred robust axonal growth and protection upon sensory neurons *in vitro*.

Schwann cells (SCs) are primary glial cells of the PNS that wrap around axons of motor and sensory neurons to form the myelin sheath 35, 36. In peripheral nerve injury, SCs are activated, become proliferating repair cells, and secrete neurotrophic factors, including NGF, BDNF, GDNF, and FGF, promoting growth cone sprouting and axonal growth and guidance 9, 37, 38. We investigated whether the CH02 peptide affected the proliferation or migration of SCs, as they are key features of SC repair 35, 39. Primary SCs were dissociated from the spinal nerves of neonatal Sprague-Dawley rats and identified by immunostaining for S-100 protein, a marker of SCs (Figure S2A). Cell viability results revealed that CH02 treatment mildly increased SC proliferation (Figure S2B). CH02-induced SC proliferation was confirmed by immunofluorescence using an antibody against the proliferating cell nuclear antigen (Figure S2C-D). Recombinant heregulin-1β1 (HRG1-β1) protein was used as a positive control. Also, CH02 peptide treatment boosted SC migration, as determined by the Boyden chamber assay (Figure S2E-F).

Collectively, these results suggested that the CH02 peptide increased the proliferation and migration of SCs.

### CH02 peptide markedly enhances axon regeneration and sensory-motor behavioral recovery after dorsal root crush injury in rats

**Figure 4:**
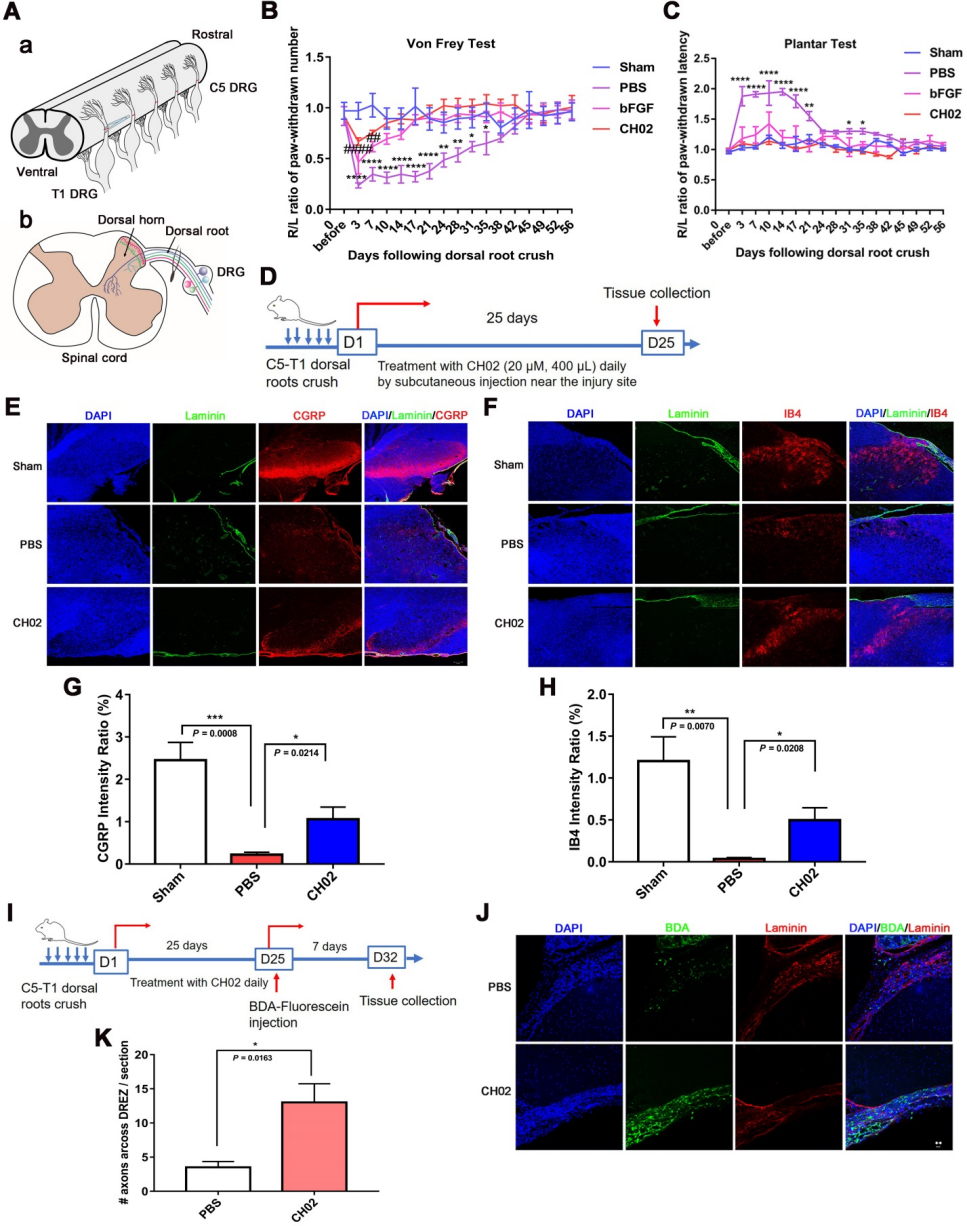
** CH02 peptide treatment after dorsal root crush injury improves axon regeneration and sensory function recovery. (A)** Procedure of the dorsal root crush model in adult Sprague-Dawley rats. Connective tissue and muscles were removed to expose the right spinal segments from the fourth cervical (C4) to the second thoracic (T2) levels. After a C5 to T1 dorsal laminectomy and dura matter opening, the right C5 to T1 dorsal roots were fully exposed. Each root was crushed three times (5s per crush) using no. 7 forceps between the dorsal root ganglia and DREZ. **(B, C)** Effect of CH02 treatment on sensory behavioral recovery in rats after dorsal root crush injury. The functional sensory recovery of rats was assessed using the von Frey test (B) and plantar test (C) at the indicated time points. Behavioral results showed that, compared to the PBS group, the recovery of pressure and thermal sensation in the CH02 and FGF2 groups was significantly improved (n = 5 for each condition; *sham vs. PBS; #sham vs. bFGF; *p < 0.05, **p < 0.01, ****p < 0.0001, ##*p* < 0.01, ####*p* < 0.0001 by two-way ANOVA with Dunnett's test; mean ± SEM). **(D-H)** Effect of CH02 treatment on axon densities in the dorsal horn of the spinal cord. **(D)** Experimental timeline: adult Sprague-Dawley rats were subjected to concurrent C5 -T1 dorsal root crush injury, followed by daily treatment with the CH02 peptide (20 μM, 400 μL) via subcutaneous injection near the injury site. After 25 days, the rats were euthanized for spinal dorsal horn tissue collection. **(E, F)** Representative fluorescence images of immunostaining for CGRP (E) or IB4+ (F), and laminin in spinal dorsal horn tissues collected from Sprague-Dawley rats 25 days after dorsal root injury. Laminin (green); CGRP and IB4+ (red); DAPI (blue). Scale bar = 50 μm. **(G, H)** Quantification of immunofluorescence intensity for CGRP (G) or IB4+ (H) from (E, F) (n = 4 animals per group; *p < 0.05, **p < 0.01, ***p < 0.001 by unpaired t-test; mean ± SEM). **(I-K)** CH02 peptide treatment enhanced axon regeneration across the DREZ. **(I)** Experimental timeline: Sprague-Dawley rats were traced with 10% biotinylated dextran amine (BDA) (0.8 μL) 4 weeks after injury. After 7 days of BDA labeling, the rats were euthanized to harvest spinal cord tissues with attached dorsal roots. **(J)** Representative images of BDA-labeled regenerated axons observed in transverse spinal cord tissue sections containing the dorsal root and DREZ. Transganglionic tracing with BDA. BDA (green); Laminin (red); DAPI (blue). Scale bars = 20 μm. **(K)** Quantification of axonal regeneration across the DREZ from (J) (n = 4 animals per group; *p < 0.05 by unpaired t-test; mean ± SEM).

We established a dorsal root injury (DRI) model to examine the effect of the CH02 peptide on axon regeneration and sensory function recovery. The right C5 to T1 dorsal roots of rats were fully exposed, and each root between the DRG and dorsal root entry zone (DREZ) was crushed three times (5s per crush) to ensure complete injury (Figure 4A). Behavioral performance was assessed using two different sensory function tests, Von Frey and Plantar. The results showed that, compared to the control group, the pressure and thermal sensation recovery was markedly ameliorated in rats treated with the CH02 peptide (Figure 4B-C). We further tested whether CH02 promoted axonal regeneration into the spinal cord. The heavy neurofilament protein NF200, the neuropeptide calcitonin gene-related peptide (CGRP), and isolectinB4 (IB4 conjugates) were used to visualize myelinated, unmyelinated peptidergic, and unmyelinated 'peptide-poor' fibers, respectively. Together, these three markers specifically identify almost all DRG neurons 40-42. We evaluated sensory axon regeneration using CGRP and IB4. Laminin is highly expressed in the dorsal root but not in the DREZ, where the dorsal root joins the spinal cord 43. We also stained these sections for laminin to definitively determine the boundary of the DREZ, which was immuno-negative for laminin. After dorsal root crush injury, rats were treated daily with CH02 peptide (20 μM, 400 μL) by subcutaneous injection near the injury site. Spinal cord tissues were collected after 25 days for immunohistochemical analysis (Figure 4D). The results revealed significantly increased CGRP- and IB4-immunoreactive axon densities in the spinal cord dorsal horn in rats treated with the CH02 peptide (Figure 4E-H). We also traced regenerated fibers by injecting biotinylated dextran amine (BDA) conjugated with fluorescein, an anterograde tracer, into the middle trunk divided from the C7 dorsal root 7 days before the rats were euthanized (Figure 4I). Compared to the PBS group, the number of BDA-positive fibers crossing into the DREZ was significantly increased in the CH02 treatment group (Figure 4J-K). Thus, CH02 exhibited the potential to promote axon regeneration and sensory function recovery.

### CH02-induced activation of FGFR signaling promotes nerve regeneration

We tested the effect of FGFRs on axon outgrowth of sensory neurons. Since CH02 showed a higher binding capacity with FGFR1 and FGFR2 than FGFR3 and FGFR4 (Figure 1D-G), we conducted transient FGFR1 or FGFR2 knockdown using small interfering RNA (siRNA) directed specifically to primary DRG neurons. We verified that three specific FGFR1 or FGFR2 siRNA constructs were effectively taken up by neurons, suppressing the mRNAs (Figure S3A-B). The scrambled sequence control siRNA had no effect. Isolated neurons were exposed to FGFR1, FGFR2, or scrambled siRNA for 12-24 h, and their neurite outgrowth patterns were analyzed. Neurons treated with FGFR1 or FGFR2 siRNA exhibited an overall reduction in neurite outgrowth (Figure S3C-D). Collectively, these findings indicated a robust role for FGFR in promoting axon outgrowth.

**Figure 5:**
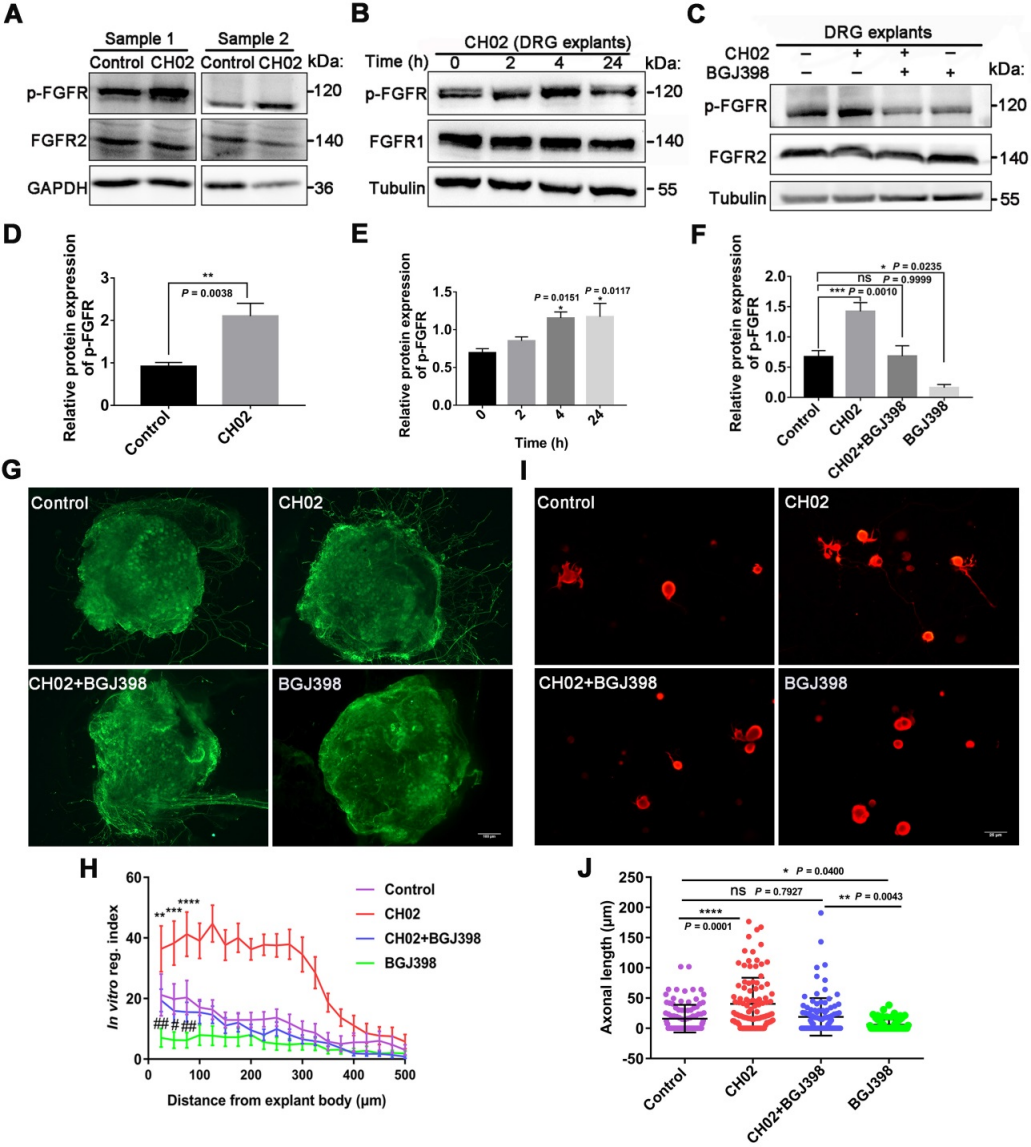
** Role of FGFR signaling in response to the CH02 peptide on promoting nerve regeneration. (A)** Western blot analysis of phosphorylated FGFR expression in C5-T1 DRGs subjected to dorsal root crush injury, followed by CH02 treatment (20 μM). DRGs were collected after 3 days. Glyceraldehyde-3-phosphate dehydrogenase (GAPDH) antibodies were used as the loading control. **(B)** Western blot analysis of phosphorylated FGFR expression in *ex vivo* cultured DRG explants of adult Sprague-Dawley rats in response to CH02 treatment at different induction times. **(C)** Western blot analysis of DRG explants treated with DMSO (control), CH02 (20 μM), CH02+BGJ398, or BGJ398 (2 μM) for 4 h to examine phospho-FGFR levels after 6 days in* ex vivo* culture. Tubulin antibodies were used as the loading control. **(D-F)** Quantification of p-FGFR expression levels in panels A, B, and C. n = 3 independent experiments. Relative protein expression levels were quantified after normalization to FGFR2 or FGFR1. Values are means ± SEM. **(G)** Representative images of the CH02 effect on axonal growth in *ex vivo* DRG explants cultured for 1 day and treated with DMSO (control), CH02 (20 μM), CH02+BGJ398, or BGJ398 (2 μM) for 4 days. DRG explants were fixed in 4% paraformaldehyde and immunostained for NF200 antibodies. Scale bar = 100 μm. **(H)** Quantification of axon growth in DRG explants (G) using Neurite-J software (n = 5 for each condition; *control vs. CH02; #control vs. BGJ398; **p < 0.01, ***p < 0.001, ****p < 0.0001, #*p* < 0.05, ##*p* < 0.01 by two-way ANOVA with Dunnett's test; mean ± SEM). **(I)** Representative images of the CH02 effect on axonal growth in primary cultured DRG neurons treated with DMSO (control), CH02 (20 μM), CH02+BGJ398, orBGJ398 (2 μM) for 48 h after 12- h of culture. Subsequently, DRG neurons were fixed in 4% paraformaldehyde and immunostained with β-tubulin antibodies. Images of random neurons were acquired by fluorescence microscopy. Scale bar = 25 μm. **(J)** Quantification of the longest axon length of each neuron randomly selected from (I) using Image-Pro Plus 6.0 software (n = 100 for each condition; *p < 0.05, **p < 0.01, ****p < 0.0001 by one-way ANOVA with Dunnett's; ns, not significant; mean ± SD).

Given the previous findings (Figs. 1 and 2), we explored the role of FGFR signaling in response to the CH02 peptide in promoting nerve regeneration. Three days after the injury, C5 - T1 DRG tissues from rats were collected for immunoblotting analysis. We observed that phosphorylated FGFR was upregulated in response to CH02 treatment (Figure 5A and D). Immunoblotting of time-course analysis also confirmed increased phosphorylated FGFR levels in *ex vivo* cultured DRG explants by CH02 treatment (Figure 5B and E). To further verify FGFR the activation in response to the CH02 peptide, we applied selective inhibitors against FGFR signals. We found that BGJ398, an inhibitor of FGFR, counteracted CH02 effects on upregulated phosphorylated FGFR levels, demonstrating that CH02 peptide could effectively promote the activation of FGFR signals in DRG tissues (Figure 5C and F). Next, we examined neurite outgrowth in primary neurons or DGR explants with combined CH02 peptide treatment and FGFR antagonism. The results indicated that BGJ398 inhibited the enhancement of neuron outgrowth associated with CH02 treatment (Figure 5G-J). Together, these results provided ample evidence that FGFR activation by the CH02 peptide was required to promote neurite growth.

### CH02 peptide binds to multiple RTK family receptors

Due to the high homology of RTK family members 44, RTK membrane proteins may also be potential CH02 targets. We performed surface plasmon resonance (SPR) and liquid chromatography-mass spectrometry (LC-MS)/MS analyses to detect the receptors affected by the CH02 peptide. Cell membrane protein samples extracted from DRG tissues were applied to the 3D light-crosslinked sensor chip immobilized with the CH02 peptide solution. Simultaneously, SPR detection was performed to monitor the binding of protein targets in real-time. The proteins bound on the chip were then digested using trypsin *in situ*, and the obtained peptides were identified by LC-MS/MS (Figure S4A). SPR detection results demonstrated an effective and reliable method (results not shown), and 21 common proteins from two biological replicate samples (Figure S4B) were obtained. According to MS scoring criteria, scores >1000, 200<score<1000, and 100<score<200 represent high-affinity, medium-affinity, and low-affinity binding, respectively. Consistent with previous results (Figure 1D-G), analysis of MS scoring results revealed that the CH02 peptide had high-affinity binding for FGFRs (FGFR1, FGFR2, and FGFR4) (Figure S4C); no FGFR3 signal was detected, likely due to its very low expression in DRG tissues (Figure S4C-D).

Besides FGFRs, other RTKs, such as hepatocyte GF receptor (MET), tyrosine-protein kinase erbB-2 (ERBB2) receptor, vascular EGF receptor 2 (VEGFR2), and platelet-derived GF receptor beta (PDGFRβ) were also captured using this method, and the relative quantitative results indicated low VEGFR2 and PDGFRβ expression in the RTK family in the DRG tissue (Figure S4C-D). We also detected the target receptor bound by the CH02 peptide in HUVEC membrane proteins using SPR and LC-MS/MS. The results demonstrated that in addition to FGFRs, CH02 bound to MET, ERBB2, VEGFR2, and PDGFRβ (Figure S5A-C). We next investigated the activation of other captured RTK members by the CH02 peptide. Immunoblotting of the time-course analysis revealed that VEGFR2 and MET phosphorylation increased in response to CH02 treatment in cultured HUVECs, but phosphorylated ERBB2 levels were downregulated (Figure S4E-J). Also, CH02 did not influence PDGFRβ phosphorylation (Figure S4F and J). These results indicated that the CH02 peptide bound to other RTK members besides FGFR, and expression of these proteins in DRG tissues was relatively low.

### Phosphoproteomic analysis of intracellular signaling pathways used by the CH02 peptide to promote nerve regeneration

**Figure 6:**
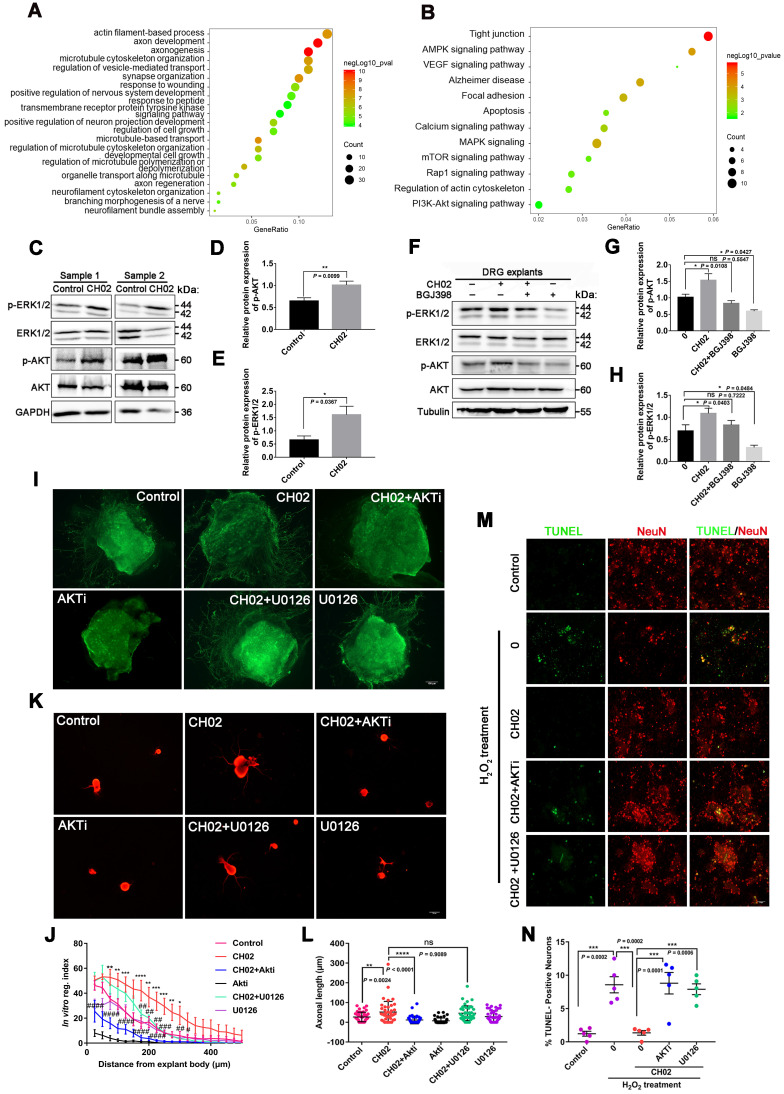
** Phosphoproteomic profiling of the intracellular signaling pathway used by CH02 for augmenting nerve regeneration. (A)** GO biological process analysis of proteins corresponding to differentially expressed phosphorylated peptides. Ordinates indicate the enriched GO terms; abscissae represent the gene ratio; size of the dots indicates the number of proteins enriched in this biological process. **(B)** KEGG pathway analysis of proteins corresponding to differentially expressed phosphorylated peptides. Ordinates indicate the enriched pathway term; abscissae represent the gene ratio; size of the dots indicates number of proteins enriched in this pathway. **(C)** Immunoblot showing phosphorylated ERK1/2 and AKT expression in C5-T1 DRGs subjected to dorsal root injury following CH02 (20 μM) treatment. DRG samples were collected after 3 days. **(D-E)** Quantification of p-ERK1/2 and p-AKT expression levels. n = 3 independent experiments. Relative protein expression level was quantified after normalization to the corresponding total proteins. Values are means ± SEM. **(F)** Immunoblot showing phosphorylated ERK1/2 and AKT expression in *ex vivo* cultured DRG explants treated with DMSO (control), CH02 (20 μM), CH02+BGJ398, or BGJ398 (2 μM) for 4 h. **(G-H)** Quantification of p-ERK1/2 and p-AKT expression levels. n = 3 independent experiments. Relative protein expression level was quantified after normalization to the corresponding total proteins. Values are means ± SEM. **(I)** Representative images of the CH02 effect on axon growth in DRG explants treated with DMSO (control), CH02 (20 μM), CH02+AKTi, AKTi (2 μM), CH02+U0126, or U0126 (2 μM) for 4 days. The axon is shown in fluorescence (NF200) images. Scale bar = 100 μm. **(J)** Quantification of axon growth in DRG explants (I) using Neurite-J software (n = 4 for each condition; *control vs. CH02; #CH02 vs. CH02+AKTi, CH02+U0126; *p < 0.05, **p < 0.01, ***p < 0.001, ****p < 0.0001, #*p* < 0.05, ##*p* < 0.01, ###*p* < 0.001, ####*p* < 0.0001 by two-way ANOVA with Dunnett's test; mean ± SEM). **(K)** Representative images showing CH02 effect on axonal growth of DRG neurons treated with DMSO (control), CH02 (20 μM), CH02+AKTi, AKTi (2 μM), CH02+U0126, or U0126 (2 μM) for 48 h after 12 h of culture. For quantification of axon length, neurons were immunostained using an antibody against β-tubulin. Images were acquired from random neurons for further analysis. Scale bar = 50 μm. **(L)** Quantification of the longest axon length of each neuron randomly selected from (K) using Image-Pro Plus 6.0 software (n = 40 for each condition; **p < 0.01, ****p < 0.0001 by one-way ANOVA with Dunnett's test; ns, not significant; mean ± SD). **(M)** Representative TUNEL staining of primary cultured apoptotic neurons induced by H_2_O_2_ for 12 h after pretreatment with DMSO (control), CH02, CH02+AKTi, or CH02+U0126for 24 h. Scale bars = 50 μm. TUNEL (green); NeuN (red). **(N)** Quantification of TUNEL-positive neurons (M) using ImageJ software (n = 5 for each condition; ***p < 0.001 by one-way ANOVA with Dunnett's test; mean ± SEM).

We next determined the intracellular signaling pathways by which CH02 promoted nerve regeneration. We performed large-scale, relative quantitative phosphoproteomic analysis in three independent biological replicates of DRG tissues with attached dorsal roots after crush injury in rats using a TiO2 enrichment strategy (Figure S6A). We identified 5078 phosphorylated sites and 3355 phosphorylated peptides in 1669 proteins (Figure S6B and databases S1 and S2). Distribution analysis of all phosphorylation modification sites showed that 59.03% of the proteins had multiple (≥2) modification sites, with as many as 123 modification sites on the P15205 protein (Figure S6C). Principal component analysis revealed a difference between the CH02 and PBS treatment groups across datasets (Figure S6D). Phosphorylated peptides screened using the > 1.2-fold change and p< 0.05 criteria were significantly differentially expressed. Phosphoproteomic analysis demonstrated 464 phosphorylated peptides to be differentially expressed between the control and CH02 treatment groups with 234 upregulated and 230 downregulated (Figure S6E-F and databases S3). Gene ontology (GO), biological process analysis of proteins corresponding to differentially expressed phosphorylated peptides, revealed enrichment of most axon growth-related cellular functions, such as axon development, axonogenesis, regulation of microtubule cytoskeleton organization, regulation of cell growth, positive regulation of nervous system development, response to wounding, and axon regeneration, suggesting that the CH02 peptide was capable of reactivating axon growth programs (Figure 6A). Furthermore, the transmembrane receptor protein tyrosine kinase signaling pathway was significantly enriched, indicating that RTK signaling may be the primary upstream pathway used by the CH02 peptide. Kyoto Encyclopedia of Genes and Genomes (KEGG) pathway analysis identified enrichment in pathways, most of which are involved in regulating axon outgrowth or regeneration, including AMPK 41 and MAPK signaling 14, 45-47, regulation of actin cytoskeleton 48, 49, and calcium 3, 50, mammalian target of rapamycin (mTOR) 6, 51-54, VEGF 55-58, and PI3K-Akt signaling (Figure 6B) 8, 59, 60. Of note, several of these signaling pathways are directly or indirectly regulated by FGFRs or other RTK signals 22, 61-65. In addition, the MAPK, PI3K, and calcium signaling are classical intracellular pathways downstream of FGFRs.

Together, these results revealed that the CH02 peptide affected phosphorylation levels of various proteins related to the axon growth program, and RTK downstream signals were significantly enriched.

Based on our phosphoproteomic data (Figure 6A-B and Figure S6) and similar studies by other investigators 3, 8, we examined whether CH02 functions by activating AKT or ERK signaling downstream of FGFRs. Immunoblot analysis revealed phosphorylated ERK1/2 and AKT expression in DRGs subjected to DRI followed by CH02 treatment for 3 days. A significant increase in phosphorylated ERK1/2 and AKT expression levels was observed, suggesting activation of these signaling pathways (Figure 6C-E). We applied an FGFR inhibitor, BGJ398, to confirm whether CH02 activated AKT and ERK upstream of FGFR and detected phosphorylated ERK1/2 and AKT levels in DRG explants treated with the CH02 peptide for 4 h. We found that the BGJ398 inhibitor counteracted CH02 effect on upregulating phosphorylated AKT and ERK levels, indicating that AKT and ERK activation by the CH02 peptide depended on upstream FGFRs (Figure 6F-H).

Next, we explored whether the CH02 peptide promoted axonal growth through AKT and ERK signaling. We found that AKTi, an inhibitor of AKT, strongly reduced the axon growth-promoting effect of CH02 (Figure 6I-L), whereas U0126, an inhibitor of the ERK pathway, had little effect (Figure 6I-L). These results suggested that the increased axon outgrowth by CH02 primarily depended on regulating AKT signaling downstream of FGFRs. Since ERK and AKT as pro-survival signaling molecules were implicated in regulating neuronal survival 66-69, we determined whether CH02- mediated-enhanced neuron survival depended on the AKT or ERK signal downstream of FGFRs. We observed that both AKTi and U0126 significantly reduced the protective effect of CH02 on neurons from apoptosis induced by H_2_O_2_ (Figure 6M-N).

Collectively, these findings indicated that the CH02 peptide ameliorated nerve regeneration by activating AKT and ERK signaling downstream of FGFRs.

### CH02-induced activation of mTOR downstream of AKT signaling augments axon growth potential

**Figure 7:**
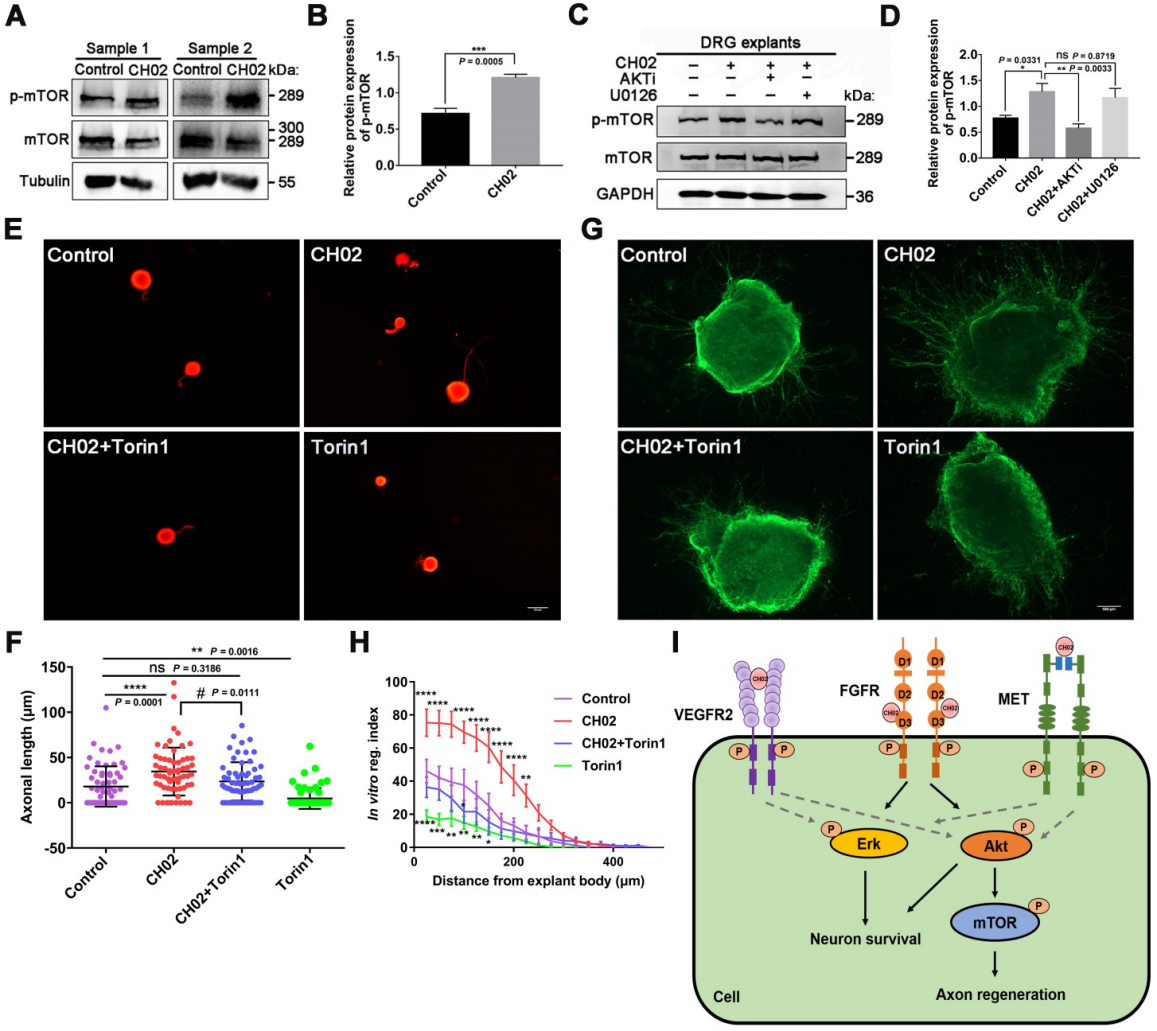
** CH02-stimulated activation of AKT/mTOR signaling is required for growth potential augmentation of axons. (A)** Immunoblot analysis showing phosphorylated mTOR expression in C5-T1 DRGs subjected to dorsal root injury followed by CH02 treatment (20 μM). DRG samples were collected 3 days post-treatment. **(B)** Quantification of p-mTOR expression levels in A. n = 3 independent experiments. Relative protein expression levels were quantified after normalization to mTOR. Values are means ± SEM. **(C)** Immunoblot showing phosphorylated mTOR expression in DRG explants pretreated with DMSO (control), CH02 (20 μM), CH02+AKTi (2 μM) or CH02+ U0126 (2 μM) for 4 h. **(D)** Quantification of the p-mTOR expression from C. n = 3 independent experiments. Relative protein expression levels were quantified after normalization to mTOR. Values are means ± SEM. **(E)** Representative images of the CH02 effect on axon growth in DRG neurons pretreated with DMSO (control), CH02 (20 μM), CH02+Torin1, or Torin1 (1 μM) for 48 h after 12-h culture. The axon is shown in fluorescence (β3-tubulin) images. Scale bar = 50 μm. **(F)** Quantification of the longest axon length of each neuron from (E) (n = 62 for each condition; *Control vs. CH02, CH02+Torin1, Torin1; # CH02 vs. CH02+Torin1; **p < 0.01, ****p < 0.0001, #*p* < 0.05 by one-way ANOVA with Dunnett's test; ns, not significant; mean ± SD). **(G)** Representative images of the CH02 effect on axon growth in DRG explants treated with DMSO (control), CH02 (20 μM), CH02+Torin1, and Torin1 (1 μM) for 4 days. DRG explants were fixed in 4% paraformaldehyde and immunostained for NF200 antibodies. Scale bar = 100 μm. **(H)** Quantification of axon growth in DRG explants from (G) using Neurite-J software (n = 4 for each condition; *control vs. CH02, CH02+Torin1, Torin1; *p < 0.05, **p < 0.01, ***p < 0.001, ****p < 0.0001 by two-way ANOVA with Dunnett's test; mean ± SEM). **(I)** Schematic illustration showing putative signaling pathways involved in CH02-mediated axon regeneration in rat sensory neurons. FGFR signal activation is the primary factor by which the CH02 peptide promotes axon regeneration. Activation of AKT/mTOR signaling in response to the CH02 peptide to enhance axon regeneration primarily depends on upstream FGFR signaling. The CH02 peptide also has an affinity for VEGFR2 and MET and can simultaneously activate VEGFR2 and MET at the cellular level. The dotted line represents unverified results.

Numerous studies have reported that mTOR activation profoundly enhances axon regeneration after CNS and PNS injuries 6, 52, 54. KEGG pathway analysis using phosphoproteomics data revealed enrichment of the mTOR pathway (Figure 6B), a downstream mediator of AKT signaling 62, 70. Hence, we analyzed whether the CH02 peptide promoted axon growth by activating the mTOR pathway downstream of AKT. Immunoblotting results showed that the phosphorylated mTOR level was upregulated in response to CH02 treatment (Figure 7A-B). Next, we applied AKT and ERK inhibitors AKTi and U0126 and detected phosphorylated mTOR expression in CH02-treated DRG explants. The data indicated that AKTi effectively reduced the CH02-induced increased phosphorylated mTOR levels, whereas U0126 had little effect (Figure 7C-D). These results demonstrated that the CH02 peptide activated mTOR through the upstream AKT signaling. We applied the combination of CH02 and the selective mTOR inhibitor Torin1 to verify whether CH02 strengthened axonal growth through mTOR signaling and examined neurite outgrowth in primary neurons or DGR explants. We observed that Torin1 greatly reduced the effect of the CH02 peptide in promoting axon growth (Figure 7E-H).

We concluded that the CH02 peptide increased axon outgrowth by increasing mTOR phosphorylation downstream of AKT.

## Discussion

The current treatment strategies for nerve injury repair have made many advances in addition to conventional methods, such as surgical intervention, stem cell therapy, and other new methods 71. However, the application of these methods is still limited by the repairability of the neurons. Thus, treatment methods based on a variety of key nerve growth factors involved in neurodevelopmental signals are still effective strategies for promoting nerve regeneration and functional recovery. Here, using a phage display peptide library, we identified a novel FGFR-targeting small peptide, CH02, which exerted a significant therapeutic effect on augmenting nerve regeneration.

Nerve injury repair in adult mammals is a complex and difficult process that relies on the reactivation of various signals during development 4, 21, 26. FGFs and their receptors (FGFRs) have the most widespread and best-documented roles in regulating neural development and are involved in neuroprotection and repair in response to neural tissue injury 21. Axon growth promotion and nerve repair by FGFs are regulated by diverse signaling mechanisms, including PI3K/Akt, MAPK/ERK, JNK/c-Jun, and other noncanonical signal transduction pathways 72. Several studies have reported that the administration of exogenous recombinant FGFs has a considerable effect in promoting nerve regeneration 14, 29, 30, 73, denoting FGFRs as important targets for nerve regeneration and repair. Currently, there is a need for small molecule agonists that can replace FGF for nerve injury repair.

The low molecular weight peptide discovered in our study can be used as an FGFR agonist to promote nerve injury repair. Compared to small-molecule chemical drugs, peptides have reduced toxicity, predictable metabolic characteristics, do not accumulate in human tissues, and exhibit increased biological specificity for the target. Small peptides with characteristic low molecular weight are attractive options and have more advantages than large-molecular recombinant proteins in terms of production cost and permeability at the lesion site. In this context, phage display technology is a simple but powerful tool that can identify small peptides that target a given molecule unbiasedly 74, 75. The strength of this technology is the very large size of the library, containing over 10^9^ different peptide sequences 76, 77. We employed a Ph.D.-7 library to identify a peptide, CH02, that targets FGFR2 by gradually reducing the concentration of the target protein (Table 1). Because the FGFR family has high homology, the CH02 peptide had high affinity for FGFR2 and also exhibited an affinity for other FGFR receptors (FGFR1, FGFR3 and FGFR4). Based on the ITC results, the CH02 peptide showed a higher binding affinity to FGFR1 and FGFR2 than FGFR3 and FGFR4 (Figure 1D-G). Similarly, it could also activate the FGFR signal (Figure 1H-I). The peptide library covered different combinations of seven natural amino acids. Therefore, the CH02 peptide sequence identified in this study was probably the sequence with the best binding ability to FGFR2 among the peptides displayed in the library. Also, the CH02 peptide sequences could be modified for additional functionality.

Although phage peptide library screening and ITC experiments showed the interaction between the CH02 peptide and FGFR, the action of agonists also depends on the specific binding mode of receptors. Therefore, we conducted molecular docking and dynamic simulation to explore the binding mode between the CH02 peptide and FGFR2 and whether CH02 affected the interaction between FGFR2 receptors. The MD results suggested that the D3 region of FGFR2 was the key binding site for CH02, and the residues that interacted with CH02 or the natural FGF2 ligand exhibited almost no overlap (Figure 2 and Figure S1). Furthermore, the CH02 peptide alone was sufficient to induce interactions between FGFR2 receptors during simulation. These results suggested that the interaction mode between CH02 and FGFR2 differed compared to that of the FGF2 ligand. Hence, we speculated no competitive effect of CH02 and FGF2 ligand on FGFR2 activation. This activation mode of the CH02 peptide has not been reported previously, suggesting a novel strategy for developing small molecule agonists for tyrosine kinase receptors. Furthermore, in MD simulation, CH02 exhibited an affinity for βC'-βE in the D3 region for inducing the binding of two FGFR2 receptors (Figure 2 and Figure S1). The βC'-βE fragment contributed to the structural difference between FGFR1 and FGFR2, indicating differential effects of the CH02 peptide on FGFR1 and FGFR2.

Since CH02 bound FGFRs and effectively promoted their activation, we considered it an FGFRs agonist for the treatment of nerve injury and axon regeneration. Mammalian sensory neurons with cell bodies in the DRG share unique features of CNS and PNS neurons 3 and are thought to represent an ideal system for studying mammalian axon regeneration. In this study, we focused on the CH02 effect on axon regeneration* in vivo* and *in vitro* based on the DRG model. Our results indicated that CH02 strongly promoted axon outgrowth and survival of primary sensory neurons *in vitro*, an effect that was comparable to FGF2 (Figure 3). Next, we established the DRI model to examine the CH02 effect on nerve regeneration* in vivo*. Our results revealed that the CH02 peptide robustly enhanced axon regeneration and sensory-motor behavioral recovery after DRI in rats (Figure 4). In behavioral testing, the CH02 recovery effect for improving the pressure and thermal sensations in rats was equivalent to FGF2. Since CH02 has only a 7-amino acid sequence compared to the complex structure of FGFs, it is also suitable as a lead compound for further structural modifications to adapt to the complex pathological environment. Besides, CH02 is composed of naturally occurring amino acids, making it more conducive to catabolism after treatment rather than accumulation with few side effects. In summary, the CH02 peptide effectively enhanced the regeneration of sensory nerves in the PNS. The neuroprotective effect of FGFs in CNS injury has been confirmed, suggesting that CH02 peptide might be suitable for treating CNS injury.

DRG damage repair is a complex systemic process. We used phosphoproteomics to detect a panorama of signal activation in the CH02-stimulated DRG tissue. The data showed that the CH02 peptide affected the phosphorylation level of a variety of proteins related to the axon growth program, significantly enriching RTK downstream signals (Figure 6A-B). Given that the MAPK and PI3K-AKT are classic intracellular signaling pathways downstream of FGFRs, we prioritized detecting the activation of these two pathways induced by the CH02 peptide. Our results indicated that CH02 simultaneously activated AKT and ERK1/2 and that the activation of AKT and ERK signaling primarily depended on the upstream FGFRs (Figure 6C-H). Next, we applied the selective inhibitors AKTi (an inhibitor of AKT) and U0126 (an inhibitor of the ERK pathway) and further explored whether CH02 promoted axonal growth through AKT and ERK signaling. Our data indicated that ERK inhibition did not affect intrinsic axon outgrowth in dissociated adult DRG neuron cultures or adult DRG explants 78. However, we found that AKT inhibition greatly reduced the CH02 effect on promoting axon growth (Figure 6I-L). Hence, the results suggested that CH02 primarily depended on the AKT pathway downstream of FGFRs to regulate axon growth.

Consistent with previous findings that neuronal survival is mediated by PI3K/AKT and ERK pathways 68, 69, we demonstrated that the CH02 peptide depended on AKT and ERK pathways to enhance neuronal survival (Figure 6M-N). KEGG pathway analysis of the phosphoproteomics data revealed enrichment of the mTOR pathway (Figure 6B). Importantly, mTOR is a known downstream mediator of AKT signaling, and various studies have shown that its activation significantly strengthens axon regeneration after both CNS and PNS injuries 6, 53, 54. Indeed, our results showed that mTOR activation downstream of CH02-induced AKT signaling augmented the axon growth potential (Figure 7). Overall, the CH02 peptide promoted FGFR-induced axon regeneration through AKT/mTOR signaling. However, other pathways may be involved and need to be examined in the future.

Our results confirmed that FGFR activation was a prerequisite for the CH02 peptide to promote neurite growth (Figure S3 and Figure 5). However, due to the high homology of RTK family receptor sequences 44, we further examined additional targets bound by the CH02 peptide using SPR and LC-MS/MS (Figure S4-5). Our results showed that in addition to FGFRs, CH02 had an affinity for VEGFR2, ERBB2, MET, and PDGFRβ in the RTK family and could activate VEGFR2 and MET at the cellular level (Figure S4). Nerve trauma repair is a complex process involving angiogenesis, and the activation of FGFR2 and VEGFR2 is a strong angiogenic signal at the wound site, suggesting that the repair of nerve injury by CH02 is multi-dimensional.

In summary, using a phage display peptide library, we identified CH02 as a novel FGFR-targeting peptide to promote nerve injury repair. Based on the DRG model, our results revealed a significant role of the CH02 peptide in promoting axon regeneration *in vitro* and *in vivo*. Collectively, our results show a great potential of CH02 for treating peripheral and central nervous system injuries.

## Methods

### Animals

All procedures involving the use of animals were performed in accordance with the animal protocol approved by the Institutional Animal Care and Use Committee of Jinan University. Male Sprague-Dawley rats (weighing 200-220 g) were purchased from Hua Fukang Biological Polytron Technologies Inc. (Beijing, China) and were housed in the University Animal Facility.

### Cell lines and cultures

HUVECs and FGFR2-overexpressing HEK293 cells were maintained in our laboratory and cultured in Dulbecco's Modified Eagle's Medium (DMEM; Gibco, Waltham, MA, USA) containing 10% fetal bovine serum (FBS; Life Technologies, Carlsbad, CA, USA), 100 U ml^-1^ penicillin and 100 μg ml^-1^ streptomycin at 37 °C in a humidified incubator under 5% CO2. The cell lines were tested for mycoplasma contamination.

### Phage library screening

The Ph.D. -7 phage display peptide library kit (New England BioLabs, Ipswich, MA, USA) was used for biopanning. Recombinant FGFR2IIIc extracellular domain protein (Sino Biological Inc., Beijing, China) was the screening target. All procedures were performed as previously described 76. After three rounds, plaques containing the phage genome were sequenced to identify the phage peptide.

### Peptide synthesis

Peptides CH01, CH02, and CH03 were synthesized by Top-Peptide Biotechnology Co. Ltd. (Shanghai, China), and their molecular weights were determined by mass spectrometry. Their purity was ≥ 98%.

### ITC

The CH02 peptide and the extracellular domain recombinant proteins (FGFR1IIIc, FGFR2IIIc, FGFR3IIIc, and FGFR4) were dissolved in water. ITC assays were performed using the MicroCal Omega ITC200 system (MicroCal, Northampton, MA). The reference cell (200 μL) was loaded with water for all experiments. The sample cell (200 μL) was filled with the recombinant protein at a concentration of 0.002 mM, and the injection syringe (40 μL) was filled with CH02 peptide at a concentration of 0.1 mM. The stirring speed was 750 rpm at 25°C with constant pressure for all ITC experiments. In the data analysis, the titration first injection (0.4 μL) was discarded. Then, 17 drops of 2 μL each were injected into the sample cell at 5 min intervals. ITC data were evaluated with a single-site binding model utilizing Origin 7.0 software (OriginLab Corp., Northampton, MA).

### Molecular docking

For molecular docking, the structure file of the extracellular segment of FGFR2 was prepared by homology modeling. The protein sequence of the extracellular segment of FGFR2 (NP_001138387.1) was from the National Center for Biotechnology Information, and the template file of the extracellular segment of FGFR2 was from the Protein Data Bank (PDB ID: 1IIL). The homology modeling of the FGFR2 extracellular segment was performed using the SWISS-MODEL online server 79, 80. Molecular docking between the CH02 peptide and FGFR2 was performed using the LeDock software. This method was based on a combination of simulated annealing and genetic algorithms to optimize the position and orientation of the ligands, and the score was assigned based on physical and empirical methods 81. During the docking process, the entire protein surface of the extracellular segment of FGFR2 was set as the search area, and the best conformation was selected based on the docking binding energy and binding pose. The results were visualized using PyMOL and Discovery Studio 2017 clients.

### MD simulation

MD simulations were performed using the same parameters for all FGFR2s or CH02-FGFR2 complexes in the analysis. All-atom MD simulations under the CHARMM36 force field were performed using the Gromacs 2019.3 software package 82. The topology and coordinate files of the FGFR2 and the CH02 peptide were generated by the pdb2gmx program using Gromacs. Initial configurations of CH02 and FGFR2 were placed in a cubic box under periodic boundary conditions PBC and with a minimum distance of 1.2 nm between simulated objects and the simulation box in the X, Y, and Z directions. Water (Model: TIP4P) was added to the simulation box system as a solvent, and then the electrostatic charge of the system was neutralized by chloride and sodium ions. The final concentration of sodium chloride was 150 mM to simulate the physiological environment. The system energy was minimized by the steepest descent method, the convergence threshold was set to 250 kJ/mol/nm, and the maximum number of iterations was 5000. Subsequently, a position-restricted pre-equilibration simulation of 1 ns was performed to further balance the system. Velocity rescaling with a stochastic term was used for temperature coupling, and the temperature coupling constant was set to 0.1 ps. Simultaneously, the pressure at 1 bar was maintained by the Parrinello-Rahman pressure coupler and all bonds were constrained using the LINCE algorithm. The long-range electrostatic interaction calculation method was set to 1.0 nm. The cutoff values of van der Waals and long-range Coulomb forces were 1.0 nm. A single CH02-FGFR2 docking complex and two CH02-FGFR2 docking complexes were simulated for 100 ns and 200 ns, respectively. The simulation step was 2 fs, and the coordinates were recorded every 30 ps. Approximately 200 G data were collected. The RMSD value and the number of hydrogen bonds were counted using the Gromacs built-in program, and the graphs of RMSD and hydrogen bonds were plotted using GraphPad. The average structure and dynamic trajectory of the protein-ligand complex were visualized using PyMOL.

### Primary culture of rat adult DRG neurons

Culture plates and coverslips were prepared in advance coated with 0.1 mg/mL poly-L- lysine (PLL; Sigma-Aldrich, St. Louis, MO, USA) at 4 °C overnight, washed 3 times with sterile water, and dried at 37 °C before cell plating. DRGs from adult Sprague-Dawley rats (220-250 g) were harvested and digested with 3 mg/mL collagenase I (Invitrogen, San Diego, CA, USA) in a 3.5 cm^2^ dish for 90 min, followed by 0.25% trypsin-EDTA (Invitrogen, San Diego, CA, USA) for 15 min in a 37 °C CO_2_ incubator. Trypsinization was terminated by adding DMEM with 10% FBS to the dish followed by gentle dissociation of the sample using a 1 mL pipette tip. The dissociated neurons were centrifuged at 1000 rpm for 5 min, and the pellet was resuspended in the neurobasal medium (Neurobasal-A medium; Thermo Fisher Scientific Inc., Rockford, IL, USA) supplemented with 1× B27 (Invitrogen, San Diego, CA, USA), 4 mM GlutaMAX (Invitrogen, San Diego, CA, USA) and 1× penicillin -streptomycin (Invitrogen, San Diego, CA, USA). Finally, neurons were plated into 24 plates coated with PLL and cultured for 72-96 h to measure axon growth.

### Measurement of axon growth

After 3 days of culture, adult DRG neurons were fixed in 4% paraformaldehyde for 15 min for β-tubulin staining. Briefly, neurons were blocked in 5% bovine serum albumin (BSA; Sigma-Aldrich, St. Louis, MO, USA) with 0.1% Triton X-100 (Sigma-Aldrich, St. Louis, MO, USA) in PBS for 1 h. Subsequently, samples were incubated with anti-β-tubulin antibody (1:1000) at 4 °C overnight. The samples were washed with PBS and incubated with AlexaFluor-conjugated secondary antibodies (1:1000) at room temperature for 2 h. For the quantification of axon length, neurons were imaged using an Olympus BX51 fluorescence microscope at 200×, and the length of the longest axon of each neuron was measured and quantified by Image-Pro Plus 6.0 software (Media Cybernetics, Rockville, MD). Approximately 30 individual neurons were selected randomly in each experiment to acquire images and measure their axon length. Three independent experiments were performed.

### *Ex vivo* culture of DRG explants of adult Sprague-Dawley rats

DRG explants were harvested from adult Sprague-Dawley rats (220-250 g) and placed in a 3.5 cm dish containing 2 mL of ice-cold neurobasal medium supplemented with 1 × B27, 4 mM Glutamax and 3× penicillin/streptomycin (Pen/Strep). Each DRG explant was cleaned and trimmed using a blade to remove excess fibers and connective tissue. Matrigel (BD Biosciences, Bedford, MA) was diluted in ice-cold culture medium (1:1) before use. The cleaned DRG explants were plated in 12-well plates precoated with 10 µL Matrigel and incubated at 37 °C and 5% CO_2_ for 45 min, followed by the addition of 2 mL culture medium to the 12-well plates and maintained under culture conditions. After 7 days of culture, axon growth was measured. Briefly, DRG explants were fixed in 4% paraformaldehyde and immunostained with βIII tubulin or NF200 antibodies. Images at a magnification of 50X were acquired using a microscope. A regeneration index was calculated from the images using ImageJ software to measure neurite number at different distances from the DRG explant.

### LDH activity assay

Primary DRG neurons (1× 10^4^ cells/well) obtained from adult Sprague-Dawley rats were seeded in 96-well plates. After culturing for 12 h, neurons were pretreated with CH02 or bFGF for 24 h before treatment with H_2_O_2_ (300 μM) for 12 h. LDH release levels were detected using the LDH cytotoxicity detection kit (Dojindo Laboratories, Japan) according to the manufacturer's instructions. LDH activities were quantitated by measuring the absorbance at 450 nm using a microplate reader.

### TUNEL assay

Harvested primary neuronal cells from adult Sprague-Dawley rats were cultured for 12 h until adherent and then pretreated with CH02 or FGF2 with fresh medium for 24 h. Cells were treated with H_2_O_2_ (300 μM) for 4 h to induce apoptosis. Subsequently, apoptotic neurons induced by H_2_O_2_ in control, CH02, and FGF2 groups were detected using the TUNEL apoptosis assay kit (Beyotime, Shanghai, China) according to the manufacturer's instructions. Neuronal cells were immunostained for the neuronal marker with NeuN antibodies (1:1000; Abcam), and DAPI (Beyotime, Shanghai, China). Randomly selected fields (200× magnification) were examined by confocal microscopy (FV10i, Olympus, Tokyo, Japan), and the percentage of TUNEL-positive neurons was analyzed using ImageJ software (National Institutes of Health, Bethesda, MD). Three independent experiments were performed.

### Primary culture of SCs

Primary SCs were harvested from the spinal nerves of neonatal Sprague-Dawley rats. After isolation, primary SCs were purified with DMEM/F12 containing 10% FBS and 10 μM AraC (Macklin, Shanghai, China) to eliminate contaminating fibroblasts. After 48 h, the medium was replaced with fresh DMEM/F12 containing 3% FBS supplemented with 10 ng/mL HRG1-β1 (PeproTech, New Jersey Cranbury, USA), 3 μM forskolin (Sigma-Aldrich, St. Louis, MO, USA), and 1% Pen/Strep to expand SCs. The purity of SCs was determined by fixing in 4% PFA and immunostaining with S-100 protein antibodies (1:1000; Merck). Primary SCs were used only for 3-5 passages.

### Cell viability assay

Primary SCs (5× 10^3^ cells/well) harvested from newborn Sprague-Dawley rats were cultured in DMEM/F12 supplemented with 3% FBS, 10 ng/mL HRG1-β1, 3 μM forskolin, and 1% Pen/Strep in a 96-well plate for 24 h and then starved with fresh DMEM/F12 supplement with 1% FBS, and 3 μM forskolin for 12 h. After starvation, cells were treated with the CH02 peptide at different concentrations for 48 h. Then, cell viability was measured using the Cell Counting Kit-8 (CCK8, Dojindo, Kumamoto, Japan), and the absorbance was quantified by a microplate reader at 450 nm.

### Transwell migration assay

SC migration ability was assessed using a 24-well Transwell chamber (8 μm size, BD Falcon). Briefly, 100 µL of cell suspension (2.5 × 10 ^5^ cells/ml in DME/F12 medium with 0.5% FBS) were added into the upper chamber precoated with PLL, and 600 µL of DME/F12 medium (5% FBS) containing the CH02 peptide or HRG1-β1, and the chemoattractant was added to the lower chamber. After 12 h, the migrated cells were fixed in 4% PFA for 15 mins and then stained with crystal violet solution (0.2% crystal violet in 20% methanol) for 30 mins. The chamber was thoroughly washed with PBS, and the PET film was peeled off from the chamber using a sharp blade for image acquisition. Images of each well were obtained in five different fields using a microscope, and the number of cells in each image was quantified using ImageJ software.

### Dorsal root crush injury

Animals were anesthetized by intraperitoneal injection of pentobarbital sodium (40 mg/kg body weight) and local injection of 0.5% lidocaine. Under sterile conditions, a skin incision between the C4 and T2 spinous processes was made using a no. 10 scalpel blade. Subsequent surgeries were performed under a stereomicroscope. The muscles attached to the lamina were separated and removed using a surgical blade and microscissors to expose the lamina. A right hemilaminectomy on the C5-T1 vertebrae was performed using a fine rongeur to expose the right C5-T1 DRGs and associated dorsal roots. After dura mater opening, a concurrent C5 -T1 dorsal root crush injury was performed using no. 7 forceps. The tines of the forceps were inserted above and below the root, halfway between the dorsal root ganglia and DREZ. Each root was crushed three times, five times each, to ensure that the dorsal roots were crushed to sever all the axons within the root completely. Sham surgery was performed in the same way, but the roots were not crushed. Finally, the muscle and the skin were sutured with 6-0 and 5-0 suture materials, respectively.

### Behavioral analyses

Animals treated with CH02 or FGF2 (n = 5 animals per group) were assessed using the following behavioral tests:

#### Von Frey test

All animals were acclimated to the observation arenas for at least 60 min prior to obtaining a baseline score. Behavioral testing was conducted before surgery to obtain the baseline responses, and after dorsal root crush injuries, the tests were performed twice a week until 8 weeks post-injury.

After an acclimation period, the animal was stimulated using a Von Frey-type 0.5 mm filament, which was placed below the center of the plantar surface of the tested paw with increasing pressure. On every test day, each foot (left front and right front) was measured 5 times at 5-min intervals, and the number of paw withdrawals was recorded.

#### Plantar test

Animals were acclimated to the testing room for at least 60 min before baseline testing, conducted before any surgical procedure. After dorsal root crush injuries, temperature sensation was measured twice a week for 8 weeks. The heat source was positioned under the glass floor beneath the right or left forepaw and the plantar surface center of the tested paw was stimulated with a noxious infrared light beam until the animals withdrew their paws. Simultaneously, the paw withdrawal latency was recorded. Measurements were taken 3 times at 15-min intervals, and the heat source was automatically shut off at 20 s to avoid inducing tissue damage.

### Immunohistology, imaging, and analysis

One month after dorsal root crush injury, rats were euthanized with pentobarbital sodium and perfused with 4% PFA. Under a stereomicroscope, spinal cords with attached roots and DRG tissues were collected from perfused rats. Subsequently, tissues were postfixed in 4% PFA for 24 h, and cryoprotected in 30% sucrose (wt/vol) for at least 48 h at 4 °C. The tissues were then embedded in OCT (Sakura, Finetek, USA), cryosectioned using a cryostat, and mounted onto slides. Spinal cord and DRG tissues were cryosectioned at thicknesses of 25 µm and 15 μm, respectively. After blocking in 5% BSA with 0.1% Triton X-100 for 1 h, sections were incubated with primary antibodies at 4 °C overnight. Secondary antibodies were applied for 2 h at room temperature. The following primary antibodies were used: mouse anti-CGRP (1:1000; Abcam); rabbit anti-laminin (1:2000; Abcam); and IB4 conjugated with DyLight 649 (1:500; Vector). The corresponding secondary antibodies were used as follows: Alexa Fluor 488-conjugated anti-rabbit IgG antibody (1:1000; Abcam) and Alexa Fluor 594-conjugated anti-mouse IgG antibody (1:1000; Abcam). Images were acquired by confocal microscopy (Zeiss 710) and analyzed using ImageJ software.

### BDA tracer injections

BDA conjugated with fluorescein (Vector, Burlingame, CA, USA), an anterograde tracer, was injected for axonal tracing and to visualize the regenerating axons. Briefly, 0.8 µL of 10% BDA was manually injected slowly into the middle trunk divided from the C7 dorsal root using a microliter syringe (Hamilton, Switzerland) fitted with a 33-gauge short needle 5 weeks after injury. Rats were fixed by perfusion with 4% paraformaldehyde 7 days after BDA injection in the middle trunk, and the C7 dorsal root was harvested, sectioned, and imaged for further analysis of axon regeneration.

### siRNA transfection

Primary DRG neurons were seeded in a 6-well plate and cultured in a neurobasal medium supplemented with 1× B27, and 4 mM GlutaMAX. For siRNA-mediated knockdown of FGFR1 or FGFR2, neurons were transfected with 50 nM of either the targeting or control siRNA using Lipofectamine RNAiMAX (Thermo Fisher Scientific Inc., Rockford, IL, USA), according to the manufacturer's instructions. The siRNA sequences targeting FGFR2 were as follows:

- si-r-FGFR2_001: CCACAATGAGGTGGCTAAA; si-r-FGFR2_002: CTACCACCTTGATGTTGTT; and si-r-FGFR2_003: CCAGGGATATCAACAACAT.

The siRNA sequences targeting FGFR1 were as follows:

- si-r-FGFR1_001: GCACCAAGAAGAGCGACTT; si-r-FGFR1_002: GTAAGATCGGTCCAGACAA; and si-r-FGFR1_003: GGAAGCACAAGAATATCAT.

After 24-72 h, cells were collected for quantitative reverse transcription-polymerase chain reaction (RT-qPCR). To measure the length of axons, neurons were fixed in 4% paraformaldehyde for 15 min, for β-tubulin staining.

### RNA isolation, reverse transcription, and RT-qPCR

Total RNA was extracted from DRG neurons using RNAiso plus (TaKaRa, Dalian, China). Reverse transcription was performed using a PrimeScriptTM RT Reagent Kit with gDNA Eraser (TaKaRa, Dalian, China), according to the manufacturer's protocol. RT-qPCR was performed using SYBR ® Premix Ex Taq TM (TaKaRa, Dalian, China) in a CFX96 real-time PCR Detection System (Bio-Rad, Hercules, CA). GAPDH was used as an internal control. The sequences of the forward and reverse primers were as follows: FGFR1 forward primer: 5′-CCCCATGCTAGCTGGCGTCT-3′, FGFR1 reverse primer: 5′-GGGTTGTGGCTGGGGTTGTA-3′; FGFR2 forward primer: 5′-GGGGGAATCCAACACCCACA-3′, FGFR2 reverse primer: 5′-CAGGACCTTGAGGTAGGGCA-3′; GAPDH forward primer: 5′-TGCTGGTGCTGAGTATGTCG-3′, GAPDH reverse primer: 5′-GCATGTCAGATCCACAACGG-3′. Gene expression was evaluated by the 2 -∆∆Ct method.

### Capture of CH02 target proteins by SPR and LC-MS/MS

DRG tissues harvested from adult Sprague-Dawley rats (220-250 g) were lysed in BWLS-17 lysate, and the cell membrane proteins were extracted and the protein sample was diluted in 1x PBS (pH 7.4) to a concentration of 200 μg/mL. The CH02 peptide was dissolved at 10 mM and spotted on the designated area on the 3D light-crosslinked sensor chip (PL-CS-191037~191038, BetterWays Inc.) using a chip microarray printer (AD-1520, BioDot Corporation). The protein sample was applied to the chip at a flow rate of 2 μL/s for a duration of 260 s. After the injection, the chip was cleaned with 1x PBS (pH 7.4) to remove nonspecific adsorption (rate: 2 μL/s; duration: 260 s). The SPR technology was used to monitor the binding of protein targets that interacted with the CH02 peptide on the chip surface in real-time. Next, the proteins bound on the chip were digested with trypsin *in situ*. The obtained peptides were collected and vacuum dried. Finally, LC-MS/MS was performed for protein identification. The MS data were searched by the Mascot algorithm using Proteome Discoverer (version 1.7; Thermo Fisher Scientific Inc., Rockford, IL, USA) analysis software and the UniProtKB/Swiss-Prot protein database.

### Phosphoproteomic profiling

DRG tissues with attached dorsal roots were collected from rats treated with the CH02 peptide or PBS. Phosphoproteomic analyses were performed as previously described 83, 84. Protein samples were extracted from tissues using lysis buffer supplemented with broad-range protease and phosphatase inhibitors. Following protein quantification using the BCA protein assay, equal amounts of protein samples were digested by trypsin using the filter aided proteome preparation (FASP) method 85 followed by desalting through SepPak C18 cartridges (Waters, MA) and vacuum-drying by Speed Vac. Next, 100 μg of desalted peptides were used for each sample and labeled with TMT following the manufacturer's instructions. The phosphopeptides were enriched by titanium dioxide (TiO2) and subsequently analyzed by LC-MS/MS to determine relative phosphoproteome changes. For protein identification and quantitative analysis, MS database search was performed. MS raw files generated by LC-MS/MS were searched against the UniProt database using MaxQuant (version 1.5.5.1) software. The maximum number of missed cleavages allowed was two. Carbamidomethyl (C), TMT 6/10 plex (N-term) and TMT 6/10 plex (K) were considered fixed modifications, whereas the variable modifications were oxidation (M), acetylation (Protein N-term) and phosphorylation (S/T/Y). The cutoff false discovery rate (FDR) using a target-decoy strategy was 1% for both proteins and peptides.

### GO and KEGG enrichment analysis

Phosphorylated peptides screened using the >1.2 times fold change and p < 0.05 criteria were regarded as significantly differentially expressed. GO biological process and KEGG pathway enrichment analyses of proteins corresponding to differentially expressed phosphorylated peptides were performed. GO biological process analysis was performed using Metascape (https://metascape.org). KEGG pathway enrichment analysis was performed using KOBAS (version 3.0), and the hypergeometric test was the statistical method employed. P < 0.05 was regarded as statistically significant.

### Western blotting

DRG tissues or HUVECs were prepared using the radioimmunoprecipitation assay (RIPA) buffer containing protease inhibitor and phosphatase inhibitor cocktail and 1 mM phenylmethanesulfonyl fluoride (PMSF). Approximately 40-50 μg of protein lysate was used to perform Western blotting, as previously described 31. The primary antibodies used were as follows: rabbit polyclonal anti-phospho-FGFR (1:1000; CST); anti-FGFR2 (1:1000; Abcam), rabbit monoclonal anti-FGFR1(1:1000; CST), rabbit monoclonal anti-phospho-VEGFR2 (1:1000; CST), rabbit monoclonal anti-VEGFR2 (1:1000; CST), rabbit polyclonal anti-phospho-ERBB2 (1:1000; Abcam), rabbit monoclonal anti-ERBB2 (1:1000; Abcam), rabbit monoclonal anti-phospho-MET (1:1000; CST), rabbit monoclonal anti-MET (1:1000; Abcam), rabbit monoclonal anti-phospho- PDGFR beta (1:1000; Abcam), rabbit monoclonal anti-PDGFR beta (1:1000; Abcam), rabbit monoclonal anti-phospho-ERK1/2 (1:1000; CST), rabbit monoclonal anti-ERK1/2 (1:1000; CST), rabbit monoclonal anti-phospho-AKT (1:1000; CST), rabbit monoclonal anti-AKT (1:1000; CST), rabbit monoclonal anti-mTOR (1:1000; CST), rabbit monoclonal anti-phospho-mTOR (1:1000; CST), rabbit monoclonal anti-β-Tubulin (1:1000; CST), and rabbit monoclonal anti- GAPDH (1:1000; CST).

### Statistical analysis

Statistical analyses were performed using GraphPad Prism 7 (GraphPad Software, La Jolla, CA, USA). All results are presented as the mean ± standard deviation (SD) or standard error of the mean (SEM). One-way or two-way ANOVA with Dunnett's test was used to determine the level of statistical significance among multiple groups, and an unpaired t-test was used to determine the statistical significance between two groups. *P* < 0.05 was considered statistically significant.

## Supplementary Material

Supplementary figures and movie legends.

Supplementary databases 1.

Supplementary databases 2.

Supplementary databases 3.

Supplementary movie 1.

Supplementary movie 2.

Supplementary movie 3.

## Figures and Tables

**Table 1:** Enrichment of phages targeting FGFR2

Round	FGFR2 (μg)	Input phage (PFU)	Output phage (PFU)	Recovery (%)	Enrichment
1	10	2.0 × 10^11^	4.02 × 10^4^	2.01 × 10^-5^	1
2	5	2.0 × 10^11^	6.34 × 10^5^	3.17 × 10^-4^	16
3	2.5	2.0 × 10^11^	3.7 × 10^6^	1.85 × 10^-3^	92
