# Supplementary material for: Peptide ligands targeting FGF receptors promote recovery from dorsal root crush injury via AKT/mTOR signaling
Source: Theranostics. 2021 Nov 2;11(20):10125–47. doi: 10.7150/thno.62525 (PMC8581430; doi:10.7150/thno.62525)
Supplement: Supplementary file 1 — Supplementary figures and movie legends. [file thnov11p10125s1.pdf]

Supplementary Information

Peptide ligands targeting FGF receptors promote recovery from dorsal root crush injury via AKT/mTOR signaling

Ying Zhao[1#], Qiang Wang[1#], Chen Xie[1], Yuling Cai[1], Xue Chen[1], Yuhui Hou[2], Liu He[1], Jianping Li[2], Min Yao[3], Shuangxi Chen[2], Wutian Wu[2], Xiaojia Chen[1*], An Hong[1*]

[1]Institute of Biomedicine & Department of Cell Biology, College of Life Science and Technology, Jinan University; Guangdong Province Key Laboratory of Bioengineering Medicine; Guangdong Provincial biotechnology drug & Engineering Technology Research Center; National Engineering Research Center of Genetic Medicine, Guangzhou, Guangdong, 510632, P. R. China

[2]Guangdong-Hong Kong-Macau Institute of CNS Regeneration, Ministry of Education CNS Regeneration Collaborative Joint Laboratory, Jinan University, Guangzhou, Guangdong 510632, China

[3]School of Pharmaceutical Sciences, Health Science Centre, Shenzhen University, Shenzhen, 5 18060, China

[#]Both Ying Zhao and Qiang Wang have contributed to the work equally.

***Corresponding authors: Xiaojia Chen, tchenxj@jnu.edu.cn; An Hong, tha@jnu.edu.cn**

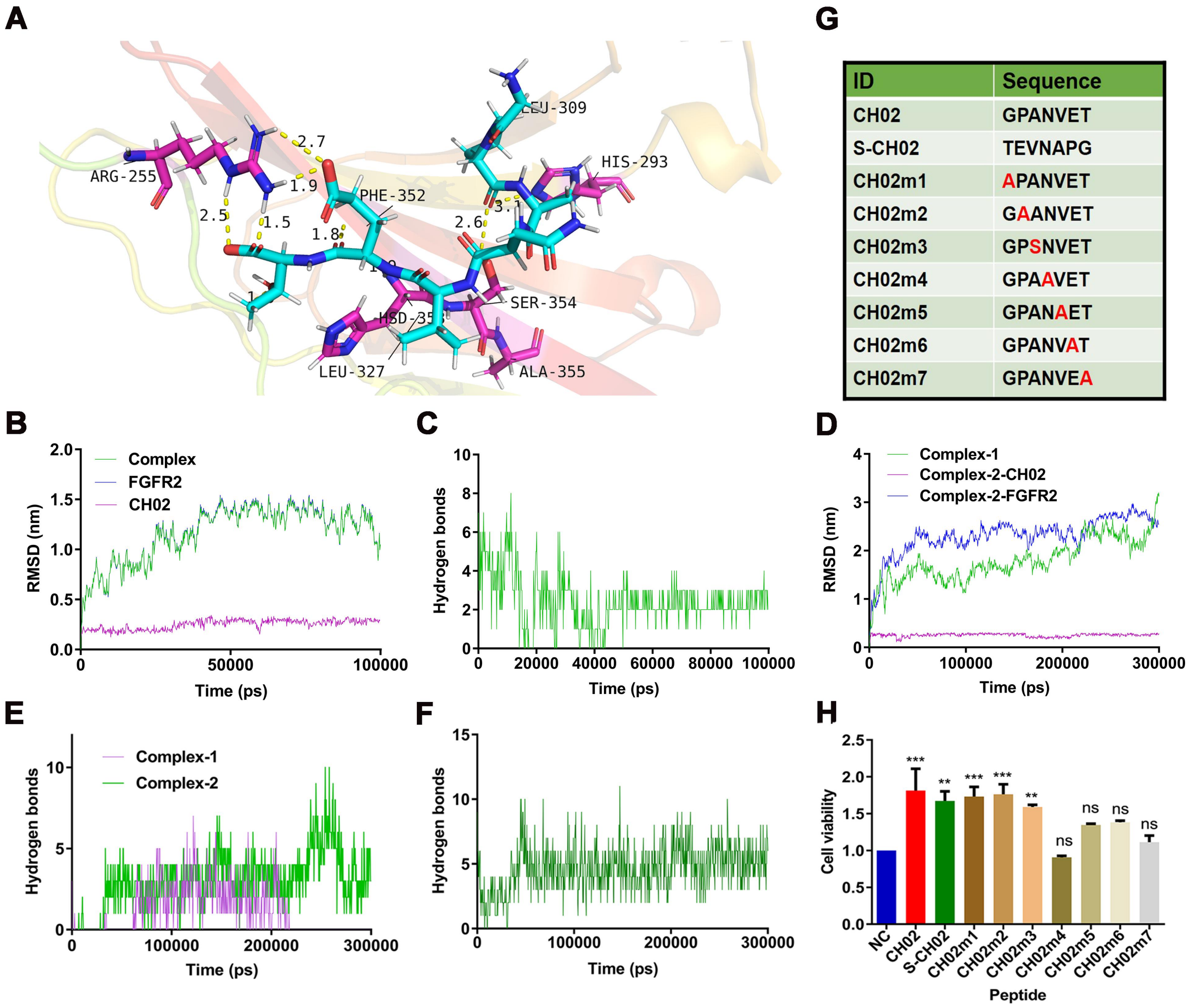

Figure S1: Molecular docking and MDS of the CH02 peptide effect on the interaction with FGFR2 receptors (related to Figure 2). (A) Three-dimensional binding pattern showing FGFR2 residues that interact with CH02. The CH02 peptide is shown as a blue stick model, partial residues of FGFR2 that interact with CH02 are shown as purple stick models, and the transparent cartoon model shows the D3 region of FGFR2. (B) RMSD values of the CH02-FGFR2 complex, CH02, and FGFR2 in the complex obtained by MDS for 100 ns. (C) Number of hydrogen bonds formed between CH02 and FGFR2 during MDS. (D) RMSD value of Complex-1, CH02 in Complex-2, and FGFR2 in Complex-2 obtained by MDS for 300 ns. (E) Number of hydrogen bonds formed between the two FGFR2 molecules in Complexes-1 and 2. (F) Number of hydrogen bonds formed between CH02 and FGFR2 in Complex- 2. (G) List of CH02 and its mutant peptide sequences. (H) Effect of CH02 and its mutant peptides on cell viability.

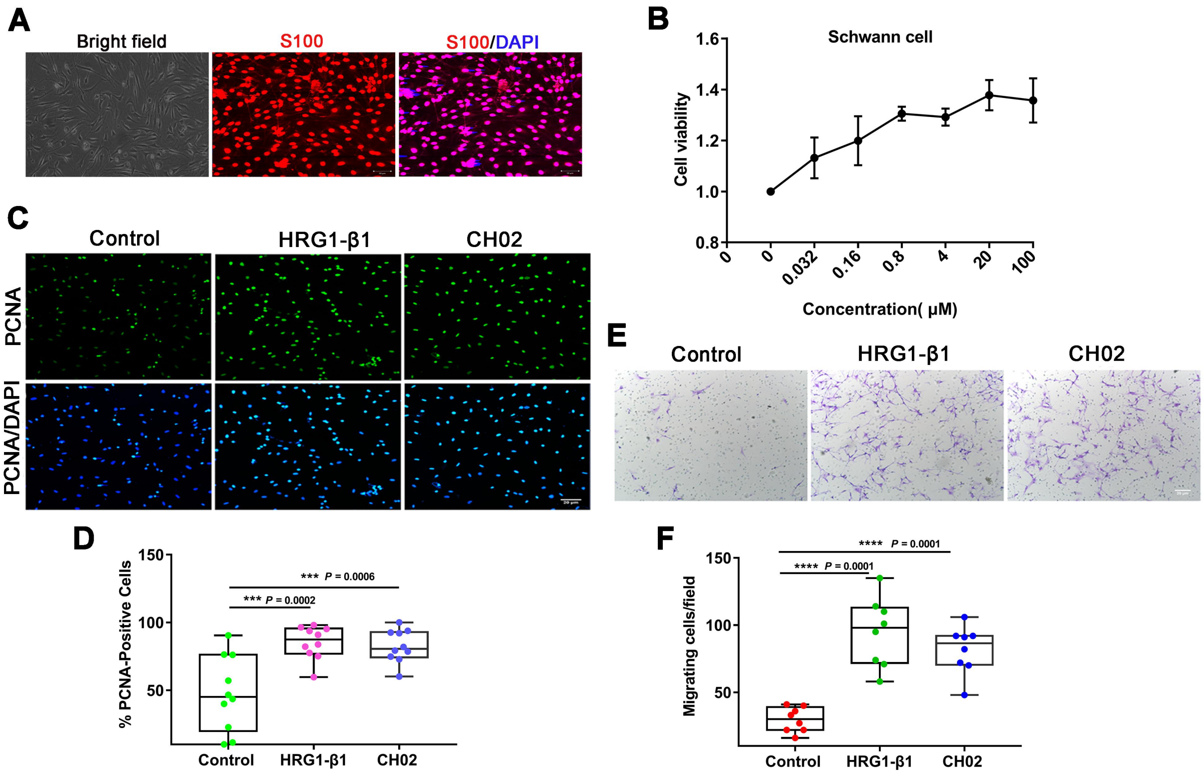

Figure S2: CH02 treatment increases proliferation and migration of primary SCs (related to Figure 3). (A) Identification of primary cultured SCs harvested from spinal nerves of neonatal SD rats. Cultured SCs exhibit bipolar or tripolar shapes under a phase-contrast microscope. Primary SCs were identified by immunostaining with the specific marker, S100. DAPI (blue); S100 (red). Scale bars = 50 μm. (B) Cell viability assay of primary cultured SCs treated with the CH02 peptide at different concentrations for 48 h. Cell viability was detected using a Cell Counting Kit-8. Values represent the mean ± SEM. (C) Representative images of the CH02 effect on the proliferation of primary cultured SCs analyzed by immunocytochemistry, using an anti-PCNA antibody (green). Recombinant HRG1-β1 protein was used as a positive control. Scale bar = 20 μm. (D) Quantification of PCNA-positive cells in (C) by ImageJ software (n =10 for each condition, ***p < 0.001 by one-way ANOVA with Dunnett's test). (E) Representative images of the CH02 effect on SC migration. Primary SCs were treated with CH02 and recombinant HRG1-β1 protein, and their migration was detected using a Boyden chamber. Scale bar = 20 μm. (F) Quantification of migrating cells (each field) from (E) (n = 8 for each condition; ****p < 0.0001 by one-way ANOVA with Dunnett's test).

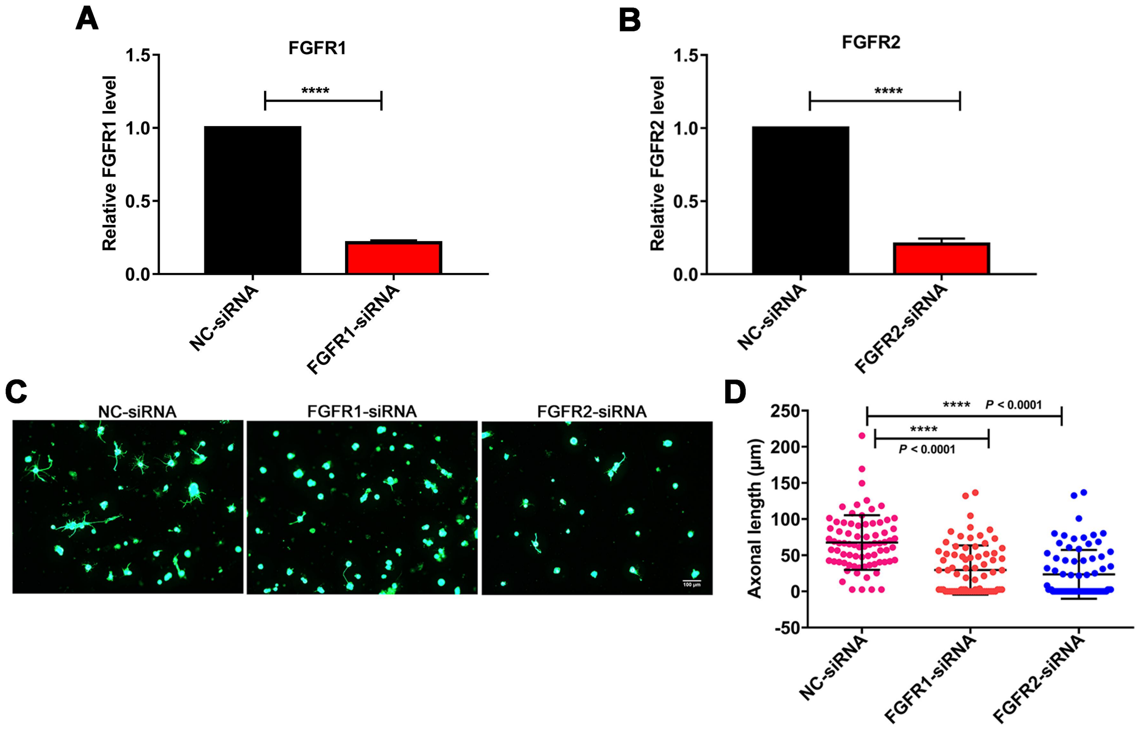

Figure S3: Transient knockdown of FGFR in sensory neurons greatly damaged axon outgrowth (related to Figure 5). (A, B) Quantitative RT-PCR showing FGFR1 and FGFR2 mRNA expression 24 h after transfection with control siRNA, FGFR1 siRNA, or FGFR2-siRNA in primary DRG neurons. Relative FGFR1 or FGFR2 expression levels in siRNA-transfected DRG neurons were quantified vs. control siRNA (****$p < 0.0001$ by unpaired t-test; mean ± SD). (C) Representative images of the knockdown effect of FGFR1 or FGFR2 on axon growth in adult primary sensory neurons. Neurons seeded in a 6-well plate were cultured for 12 h to adhere and then transfected with 50 nM of either the targeting or control siRNA for 48 h. Neurons were then fixed in 4% paraformaldehyde for 15 min for β-tubulin staining, and axon length was measured. Scale bars = 100 μm. (D) Quantification of the longest axon length of each neuron randomly selected (C) using Image-Pro Plus 6.0 software (n = 75 for each condition; ****$p < 0.0001$ by one-way ANOVA with Dunnett's test; mean ± SD).

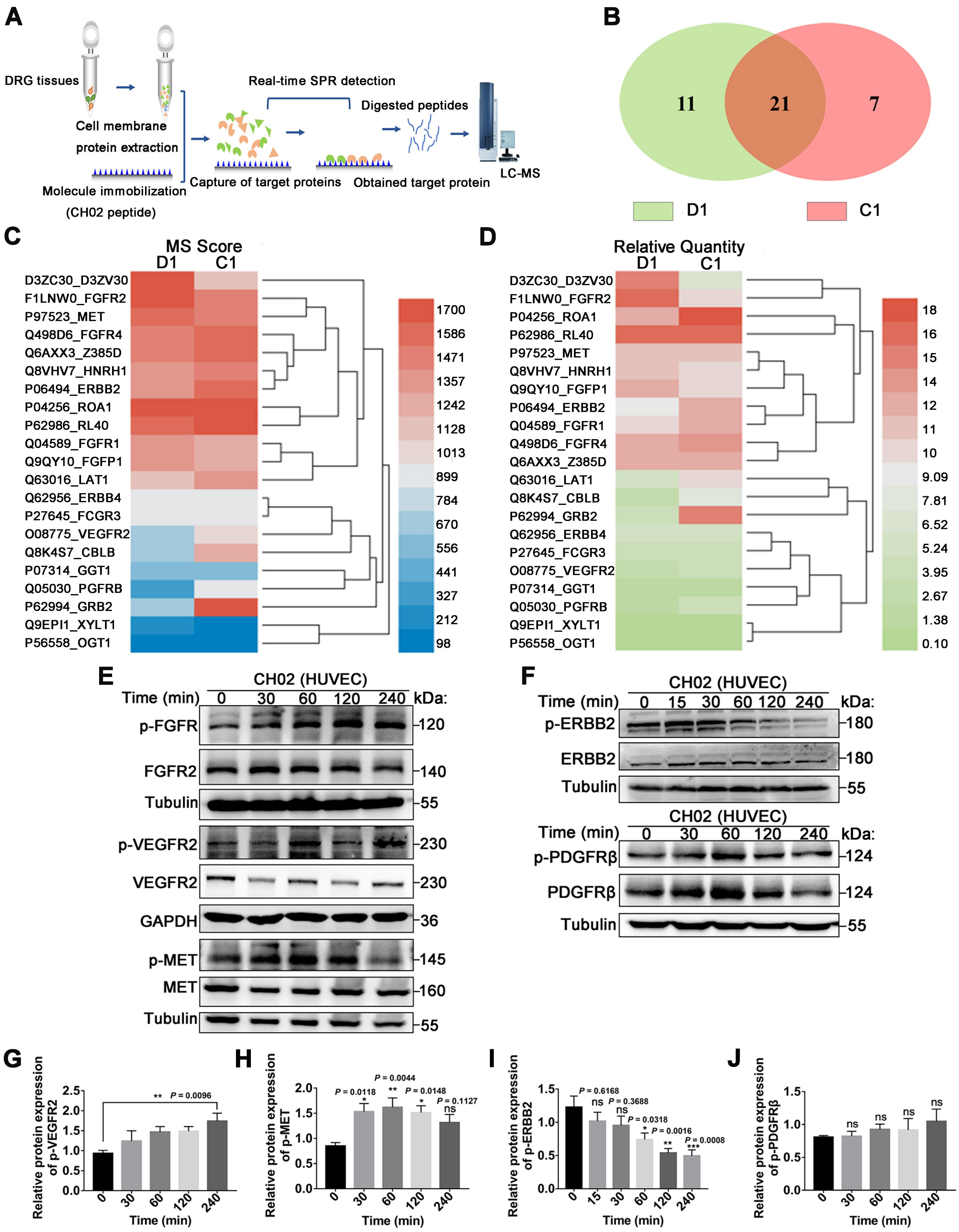

Figure S4: Identification of CH02 target receptors other than FGFRs. (A) Schematic diagram depicting the capture of CH02 target proteins. The CH02 peptide solution was spotted on the 3D light-crosslinked sensor chip by a chip microarray printer. Cell membrane proteins

from DRG tissues were extracted and diluted to a concentration of 200 μg/mL. The protein samples were applied to the chip at a flow rate of 2 μL/s (duration: 260 s). Simultaneously, SPR detection was performed to monitor the binding of protein targets in real-time. The proteins bound on the chip were digested with trypsin in situ, and the peptides were identified using LC-MS/MS. (B) Venn diagram of protein targets captured from two biological replicate samples. (C) MS score heatmap diagrams of common target proteins captured from two biological replicate groups. (D) Relative quantity heatmap diagrams of common target proteins. (E-F) Western blot analysis of phosphorylated VEGFR2, ERBB2, MET, and PDGFRβ expression in cultured HUVECs treated with CH02 at different induction times. (G-J) Quantification of p-VEGFR2, p-MET, p-ERBB2, and p-PDGFRβ expression levels. n = 3 independent experiments. Relative protein expression levels were quantified after normalization to the corresponding total proteins. Values were means ± SEM.

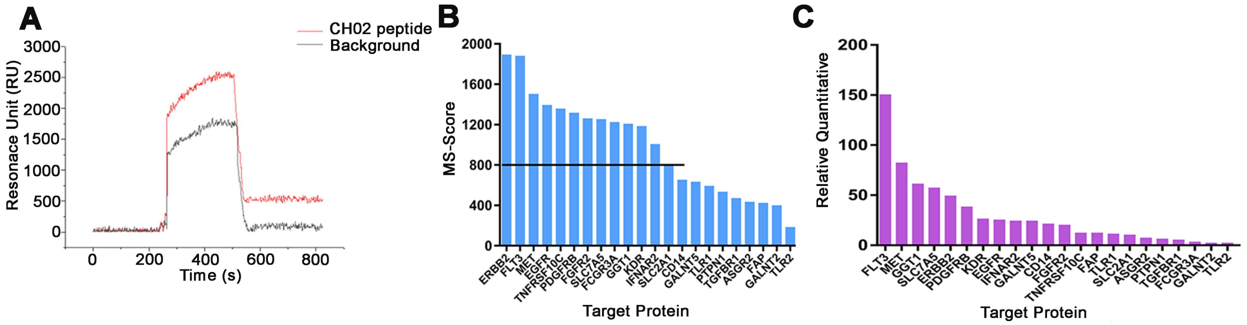

Figure S5: Identification of the target receptor bound by the CH02 peptide in HUVEC membrane proteins (related to Figure S4). (A) SPR detection result. (B) MS score histogram of target proteins captured by SPR and LC-MS/MS. (C) Relative quantity histogram of target proteins.

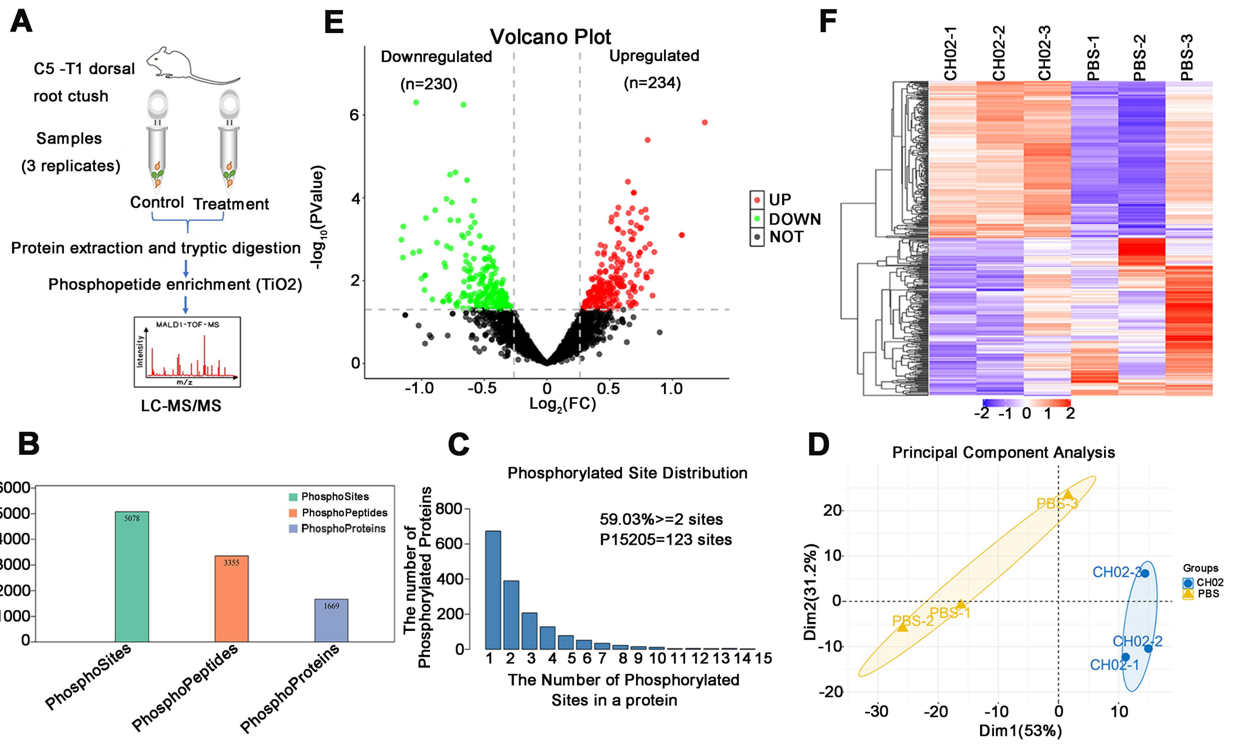

Figure S6: Phosphoproteomic profile analysis of phosphorylation site in DRG tissues with attached injured dorsal root from rats treated with the CH02 peptide or PBS (related to Figure 6). (A) Strategy for LC-MS/MS-coupled relative quantitative phosphoproteomics analysis on DRG tissues with attached dorsal roots of rats treated with CH02 peptide or PBS. (B) Phosphoproteomics identification results. A total of 5078 phosphorylation sites and 3355 phosphorylated peptides on 1669 proteins were identified. (C) Number distribution of phosphorylation sites in protein. (D) Principal component (PC) analysis of the number of phosphorylated peptides from CH02 peptide treatment group (n = 3) and PBS group (n = 3). (E) Volcano plot for differentially expressed phosphorylated peptides between CH02 peptide treatment group and control group. Differentially expressed phosphorylated peptides meet the >1.2 fold change and $p < 0.05$ criteria. Log 2 fold change is shown on the x-axis and –log 10 p-values are shown on the y-axis. The red dots represent the upregulated expressed phosphorylated peptides after treatment with CH02 peptide, whereas the green dots represent the downregulated, after treatment. (F) Heat map depicts significantly differential phosphorylated peptides. N = 3/group.

Supplementary Movie 1: Molecular dynamic simulation of the CH02-FGFR2 docking complex. FGFR2 shown as surface model, and CH02 peptide shown as sticks model.

Supplementary Movie 2: Molecular dynamic simulation of Complex-2. FGFR2 shown as

surface model, and CH02 peptide shown as sticks model. The two FGFR2s in this system were named receptors 1 and 2. The D2 and D3 domains of receptor 1 were displayed in violet and light pink, respectively, while those of receptor 2 were displayed in deep blue and sky blue, respectively.

Supplementary Movie 3: Molecular dynamic simulation of Complex-1.
